# CDKPredict: a clinicogenomic model for risk-adapted therapeutic intervention in breast cancer

**DOI:** 10.21203/rs.3.rs-10176871/v1

**Published:** 2026-09-29

**Authors:** Luca Boscolo-Bielo, Francisco Sanchez-Vega, Subhiksha Nandakumar, Antonio Marra, Enrico Moiso, Emanuela Ferraro, Ino de Bruijn, Luc Cabel, Karl Pichotta, Bianca Malagutti, Anton Safonov, Michele Waters, Julia Ah-Reum An, Mark Donoghue, Yixiao Gong, Matthew Zatzman, Maureen Carey, Sameet Sreenivasan, Gráinne Long, Sajan Khosla, Ingrid Mayer, Maurizio Scaltriti, Nicola Fusco, Elisabetta Munzone, Fresia Pareja, Dara S. Ross, Walid K Chatila, Michael F. Berger, David B. Solit, Shanu Modi, Nikolaus Schultz, Giuseppe Curigliano, Mark E. Robson, Sohrab Shah, Sarat Chandarlapaty, Pedram Razavi

**Affiliations:** 1 Department of Medicine, Memorial Sloan Kettering Cancer Center, New York, United States; 2 Translational Oncology Partnership Program, Memorial Sloan Kettering Cancer Center, New York, United States; 3 Department of Oncology and Hematology-Oncology, University of Milan, Milan, Italy; 4 Department of Epidemiology and Biostatistics, Computational Oncology Service, Memorial Sloan Kettering Cancer Center, New York, United States; 5 European Institute of Oncology IRCCS, Milan, Italy; 6 Weill Cornell Medical College, New York, United States; 7 Tempus AI Inc., Chicago, IL, United States; 8 AstraZeneca, Gaithersburg, MD, United States; 9 AstraZeneca, Cambridge, United Kingdom; 10 Department of Pathology and Laboratory Medicine, Memorial Sloan Kettering Cancer Center, New York, United States; 11 Human Oncology and Pathogenesis Program, Memorial Sloan Kettering Cancer Center, New York, United States

## Abstract

Wide variation in outcomes is observed for patients with hormone-receptor-positive metastatic breast cancer (MBC) receiving first-line CDK4/6 inhibitors plus endocrine therapy (CDK4/6i+ET). Although front-line escalation strategies are beginning to enter clinical practice, assigning patients to intensified or de-intensified approaches remains challenging due to several mechanisms of resistance spanning tumor-intrinsic and host factors. To address this, we developed CDKPredict, an interpretable machine-learning model integrating baseline clinicopathologic and genomic features to predict outcomes from first-line CDK4/6i+ET. The model was trained on a densely annotated cohort of 1,264 patients and validated in a large multi-institutional external cohort (n=564, time-dependent AUC=0.74), stratifying patients into low-, intermediate-, and high-risk groups (median progression-free survival: 29.8, 14.7, and 8.0 months, respectively; hazard ratio [high vs low]=3.85, *P*=1.5×10^−17^). The model retained robust prognostication across clinically relevant subgroups and biomarker-selected populations and outperformed the current escalation criterion of treatment-free interval ≤12 months. Notably, CDKPredict demonstrated strong clinical and biological plausibility, with high-risk tumors featuring genomic and transcriptomic programs mechanistically linked to CDK4/6i+ET resistance, and higher-risk strata progressively enriched for actionable alterations. Finally, we developed CDKPredict-S, a pragmatic and scalable model requiring a restricted number of features intended for resource-variable settings, which was validated across two independent external cohorts totaling 1,019 patients and made publicly available. Collectively, CDKPredict provides a foundation for the prospective evaluation of risk-adapted, biomarker-informed therapeutic strategies.

Risk-adapted therapeutic interventions have long been pursued in early-stage, hormone-receptor-positive/HER2-negative (HR+/HER2–) breast cancer. A cornerstone of this paradigm was the implementation of multigene assays, initially developed as prognostic tools to estimate the risk of distant recurrence and later shown to have predictive utility in guiding the use of adjuvant chemotherapy^1–3^. These approaches were subsequently refined to individualize risk estimates through integration with patient-specific clinicopathologic features^4–6^.

The implementation of similar risk-adapted strategies in metastatic breast cancer (MBC) has been challenging. Despite significant improvements in progression-free survival (PFS) and overall survival with first-line CDK4/6 inhibitors plus endocrine therapy (CDK4/6i+ET), clinical outcomes remain highly heterogeneous, with approximately 20% of patients experiencing rapid progression, and a similar proportion achieving durable disease control^7–10^. We and others have characterized a wide array of resistance mechanisms underlying this variability. Within the cell-cycle machinery, these include *RB1* loss^11^, overexpression of *CCNE1*^12,13^, aberrant Hippo (FAT1/YAP-TAZ) resulting in CDK6 overexpression^14,15^, *TP53*/*MDM2*-induced and DREAM/CDK2-mediated cell-cycle re-entry^16^, *AURKA*^17^, and *MYC* amplification driving resistance through KLHL42-mediated pRB1 degradation^18^. Other drivers of resistance to CDK4/6i and/or ET include alterations in *PTEN*, *FGFR1*, *ESR1*, *ERBB2*, *NF1*/MAPK, among other regulators of endocrine and growth-factor signaling^19–28^. Beyond individual biomarkers, several mechanisms of resistance have been identified, including germline *BRCA2*-mediated homologous recombination deficiency (HRD)^11,29^, APOBEC3 mutagenesis^30^, elevated tumor mutational burden (TMB)^30,31^, as well as certain copy-number signatures resulting in *RB1* hemizygosity as determinants of poor outcomes^11,13,32^. Collectively, these overlapping and often competing mechanisms underscore the limited power of any standalone biomarker to forecast treatment outcomes and the need for a multimodal approach for accurate baseline risk stratification.

This clinical need is further underscored by the expanding array of intensified therapeutic strategies being evaluated in the first-line setting for HR+/HER2− MBC to tackle resistant tumors. Notably, the addition of targeted therapies, such as PI3K-alpha-selective inhibitors to standard CDK4/6i+ET has proven to be effective^33^, with several ongoing phase III trials investigating such therapeutic approaches (NCT06380751, NCT06790693, NCT06065748, NCT04862663, NCT04711252, NCT04546009). However, in the absence of robust and reliable risk stratification tools, high-risk patients are currently identified using historic and crude criteria, namely treatment-free interval (TFI) ≤12 months, defined as metastatic recurrence during or within 12 months from the end of adjuvant ET, that fail to recapitulate the full array of factors promoting endocrine and/or CDK4/6i resistance. Such criteria can lead to a significant misclassification problem. Tumors fulfilling historic high-risk criteria may retain endocrine sensitivity resulting in overtreatment, health-related and financial toxicities. Conversely, biologically aggressive tumors lacking overt high-risk clinical features might be exposed to standard treatments resulting in short-term clinical benefits, treatment attrition, and poorer cancer-related outcomes.

To address this, we developed CDKPredict, an integrated clinicogenomic prognostication model trained on a large cohort of patients who received routine care at Memorial Sloan Kettering Cancer Center (MSK) and rigorously validated across multiple independent institutions and clinical settings. Our aim was to establish a pragmatic and interpretable baseline risk stratification framework to inform first-line risk-adapted therapeutic interventions in HR+/HER2− MBC.

## RESULTS

### Baseline clinical and genomic features and their prognostic associations for first-line CDK4/6i+ET

To develop a risk stratification framework, we leveraged the MSK Breast Translational Dataset, a prospectively collected and comprehensively annotated cohort of patients with breast cancer who received routine care at MSK. The final MSK-cohort comprised 1,264 patients with HR+/HER2− MBC who received first-line CDK4/6i+ET, with available baseline tumor-normal sequencing (on samples collected before and up to 28 days from treatment start) using an FDA-authorized clinical sequencing assay interrogating up to 505 cancer-associated genes^34^ (Fig. 1a, Extended Data Fig. 1).

The cohort was broadly representative of a real-world HR+/HER2− MBC population seen in a tertiary cancer center^35^, with a median age at metastatic diagnosis of 57 years (interquartile range [IQR]: 49–65), 29.1% of patients (n=368) diagnosed with de novo MBC (Fig. 1b), and visceral disease at presentation in 44.5% of cases (n=563). At a median follow-up of 50.4 months (IQR: 25.8–79.2), median progression-free survival (mPFS) was 18.6 months (95% confidence interval [CI] 17.5–20.5), with a 12-month PFS rate of 63.3% (95%CI: 60.6–66.1; Fig. 1c).

The vast majority of sequenced samples were obtained from the first metastatic biopsy (70.5%, n=891), and the genomic landscape aligned with that previously described for HR+/HER2− MBC candidates for front-line therapy^20^, with *PIK3CA*, *CCND1*, and *TP53* representing the most common alterations (Fig. 1d). We confirmed expected co-segregations between genomic and clinical features in our large cohort, including enrichment of *TP53* LoF among high-grade tumors (OR=2.85, *q*=1.8×10^−13^) and *ESR1* mutations in metastatic tumors with a TFI ≤12 months (OR=3.64, *q*=3.7×10^−06^; Fig. 1e, **Supplementary Table 1**).

Our cohort further recapitulated established prognostic biomarkers reported in prospective randomized clinical trials^9,36,37^, including shorter PFS in patients with liver metastasis (univariable hazard ratio [HR]=1.95, *q*=1.3×10^−16^; Fig. 1f), and in those with a TFI ≤12 months (univariable HR=1.69, *q*=5.2×10^−14^; Fig. 1g). Moreover, previously reported genomic alterations promoting CDK4/6i+ET resistance were significantly associated with poor outcomes (Fig. 1h), including *TP53*^16^ (HR=1.94, *q*=6.5×10^−17^) and *RB1* LoF alterations^11,13,29,32^ (HR=2.46, *q*=7.7×10^−05^; Fig. 1i, **Supplementary Table 2**).

### Development of CDKPredict: a clinicogenomic model for risk stratification on first-line CDK4/6i+ET

We next sought to develop a risk stratification model leveraging baseline clinical and genomic data for individuals receiving first-line CDK4/6i+ET (Fig. 2a). Accordingly, we split the MSK-cohort into a training set (n=884, 70%) and a hold-out validation set (n=380, 30%) using a stratified sampling approach designed to balance the rate of PFS events, presence of liver metastasis, and TFI ≤12 months, resulting in a comparable representation of clinical and genomic features between the two sets (Extended Data Fig. 2, **Supplementary Table 3**).

We developed an ensemble prediction framework comprising n=100 L1-penalized Cox models (base learners), each trained on a bootstrapped subset of the training cohort to promote feature diversity, reduce model variance and prevent overfitting^38^. A key methodological consideration was the handling of rare genomic features (such as *RB1*, *PTEN*, *BRCA1/2*, and *ESR1*), which, although established drivers of CDK4/6i+ET resistance, are present in a small minority of tumors not previously exposed to CDK4/6i+ET. Standard penalized regression tends to shrink the parameter contribution of such uncommon features toward zero, potentially obscuring clinically meaningful signals. To mitigate this, each base learner applied an adaptive penalty inversely proportional to feature importance^39^ (Methods), thereby preserving the contribution of rare but prognostically relevant alterations. The regularization terms were further optimized using a 3-fold cross-validation strategy to maximize the discriminative accuracy of the model for the time-dependent area under the curve (AUC[t]) over a 3–36 months period, reflecting a clinically meaningful and actionable timeframe for first-line therapeutic stratification in HR+/HER2− MBC. CDKPredict ultimately resulted in an aggregated risk-score ranging from 0 to 100 derived from the mean risk estimation across base learners.

We first developed a clinical-only model, incorporating baseline clinicopathological variables established to be associated with outcomes on front-line CDK4/6i+ET (**Supplementary Table 4**). In parallel, we developed a genomic-only model including oncogenic and likely oncogenic somatic and germline alterations according to OncoKB, an FDA-approved precision oncology knowledge base^40,41^, filtered for alterations present in at least 1.5% of the overall MSK-cohort (**Supplementary Table 4**). This parallel development strategy was deliberately designed to determine whether clinical and genomic features capture distinct, non-redundant prognostic signals. To guard against the risk of overconfident estimates during model development, risk scores for the training set were derived exclusively from the out-of-bag risk estimation by base learners (Methods).

In the training set, both the clinical-only (AUC[t]=0.69) and the genomic-only model (AUC[t]=0.70) demonstrated superior prognostication compared with standalone biomarkers (most discriminative clinical and genomic biomarkers: AUC[t]_TFI≤12 months_=0.62 and AUC[t]_*TP53*_=0.61; Fig. 2b–c, Extended Data Fig. 3a–b). To define clinically meaningful risk groups, we next fitted a two-component Gaussian-mixture model (GMM) to risk-scores (Extended Data Fig. 3c, Methods), identifying patients at high-risk of early progression who demonstrated poorer outcomes than those predicted at low-risk cases in both frameworks (HR_Clinical_=2.11, 95%CI: 1.79–2.47, *P*=1.4×10^−19^; HR_Genomic_=2.20, 95%CI: 1.87–2.59, *P*=9.8×10^−22^; Fig. 2d).

Evaluating whether the two models provided distinct prognostic information, we observed low agreement between the clinical and genomic risk classifications (Cohen’s kappa=0.21; Fig. 2e, Extended Data Fig. 3d). Consistently, in a multivariable Cox regression including clinical and genomic predictions, each model retained independent prognostication (Fig. 2f), emphasizing the non-redundant contribution of clinical and genomic features to predict CDK4/6i+ET outcomes. We next stratified the analyses combining clinical and genomic risks. Patients classified as clinical-low/genomic-low (i.e., predicted to be low-risk by both models) demonstrated prolonged disease control (n=356, mPFS=34.1 months, 95%CI: 29.0–44.6) compared with those classified as clinical-high/genomic-high (n=192, mPFS=7.9 months, 95%CI: 6.4–9.5). By comparison, patients with discordant risk classification demonstrated intermediate and comparable outcomes (clinical-high/genomic-low: n=150, mPFS=16.7 months, 95%CI: 13.7–21.0; clinical-low/genomic-high: n=186, mPFS=15.5 months, 95%CI: 12.6–19.4; Fig. 2g, Extended Data Fig. 3e).

Based on these results, we next embarked on the development of CDKPredict, a unified prediction model integrating clinical and genomic variables (Fig. 2h; Methods). As anticipated, the unified model demonstrated superior predictive discrimination compared with each standalone model (AUC[t]=0.74 vs. 0.69 vs. 0.70; and C-index=0.68 vs. 0.64 vs. 0.64 for the clinicogenomic, clinical, and genomic model, respectively; Fig. 2i and 2j), with cumulative risk scores showing a strong association with shorter time to progression (HR_per 10-unit increase_=1.31, 95%CI: 1.26–1.36, *P*=6.9×10^−48^; Extended Data Fig. 3f–g). Finally, leveraging our observation that the clinical and genomic models frequently captured discordant risk profiles with similar PFS, we designed the clinicogenomic model to classify patients into low-, intermediate-, and high-risk groups using a three-component GMM (Methods; Fig. 2k). Across strata, high-risk patients had significantly shorter PFS on first-line CDK4/6i+ET than those predicted at intermediate- (6.2 vs. 15.9 months, HR=2.32, 95%CI: 1.88–2.86, *P*=6.7×10^−15^) and low-risk (33.6 months, HR=4.58, 95%CI: 3.66–5.74, *P*=5.1×10^−40^; Fig. 2l).

CDKPredict performance was robustly preserved in the holdout MSK-test set (n=380; Fig. 2m, Extended Data Fig. 3h), with high-risk patients demonstrating significantly shorter PFS on CDK4/6i+ET (n=67, mPFS=8.3 months) compared to intermediate- (n=154, mPFS=16.9 months, HR=1.68, 95%CI:1.27–2.22, *P*=0.00026) and low-risk patients (n=159, mPFS=30.1 months, HR=3.91, 95%CI: 2.79–5.48, *P*=2.0×10^−15^, Fig. 2n), supporting the generalizability of the model for independent risk-prediction.

Finally, we benchmarked CDKPredict against several machine-learning architectures, including random forest, extreme gradient boosting, multilayer perceptron, residual neural networks, and a previously developed ensemble model at MSK (SCORPIO) with proven clinical validity to predict outcomes from immune-checkpoint inhibitors in a pancancer setting^42^ (Extended Data Fig. 4). These analyses showed CDKPredict to perform comparably or favorably to state-of-the-art frameworks, while retaining an architecture designed for natural clinical interpretability.

### Independent external clinical validation of CDKPredict

To obtain the independent clinical validation of CDKPredict, we next leveraged a nationwide, external, multi-institutional cohort totaling 564 patients with HR+/HER2− MBC, who received first-line CDK4/6i+ET in community or academic settings and underwent baseline tumor genomic sequencing on samples collected before and up to 28 days from first-line CDK4/6i+ET initiation (Fig. 3a, Extended Data Fig. 5). Genomic profiling was conducted using clinically validated platforms, including a tissue-based assay interrogating up to 648 cancer-related genes^43,44^ (n=417), and a 105-gene circulating tumor DNA (ctDNA) panel^45^ (n=147). Compared with the MSK-cohort, patients in the external cohort exhibited shorter PFS on first-line CDK4/6i+ET (mPFS=14.7 months, 95%CI: 12.5–17.5; Extended Data Fig. 6a), consistent with a higher representation of poor prognostic features, including a higher prevalence of liver (OR=2.06, *q*=4.7×10^−10^) and central nervous system (CNS) metastasis (OR=4.29, *q*=7.5×10^−11^), and a lower frequency of bone-only disease (OR=0.48, *q*=1.7×10^−9^; Extended Data Fig. 6b, **Supplementary Table 5**). Nonetheless, the genomic landscape of the external cohort was broadly comparable to that of the MSK-cohort (**Supplementary Table 5**).

Before deployment, the clinicogenomic model was retrained on the overall MSK-cohort, where model parameters and risk-group cutoffs were fine-tuned and locked for all subsequent evaluations (Fig. 3a). In the external cohort, CDKPredict risk-scores retained robust single-patient discrimination (AUC[t]=0.74, 95%CI: 0.68–0.77; C-index=0.65, 95%CI: 0.62–0.68; Fig. 3b), correlating with shorter PFS on CDK4/6i+ET in a stepwise manner (HR_per 10-unit increase_=1.28, 95%CI: 1.22–1.35, *P*=2.3×10^−22^; Fig. 3c).

High-risk cases demonstrated poorer outcomes on CDK4/6i+ET (mPFS=8.0 months, 95%CI: 6.4–10.0) compared with cases estimated at intermediate (mPFS=14.7 months, 95%CI: 12.3–19.5; HR=1.93, 95%CI: 1.48–2.52, *P*=1.2×10^−06^) and low risk (mPFS=29.8 months, 95%CI: 21.8–42.1; HR=3.85, 95%CI: 2.83–5.26, *P*=1.5×10^−17^; Fig. 3d). The risk stratification further resulted in pronounced differences in the rate of durable disease control at 6 months (high-risk=61.0%, 95%CI: 52.9%–70.1%; intermediate-risk=74.4%, 95%CI: 68.6%–80.8%; low-risk=84.8%, 95%CI: 80.0%–90.2%), as well as the likelihood of achieving a radiological tumor response across risk strata (*P*=0.0010 by Pearson’s chi-square test; Fig. 3e).

To ascertain the generalizability of CDKPredict to the general population of patients considered for first-line CDK4/6i+ET, we next evaluated its performance across clinically relevant subgroups. Robust model predictions were consistent across CDK4/6i compounds (*P*_Interaction_=0.93) and ET backbone (i.e., fulvestrant vs. aromatase inhibitors, *P*_Interaction_=0.34; Fig. 3f, **Supplementary Table 6**). Moreover, while the model was not trained on patients with baseline ctDNA profiling, it retained strong risk stratification on the subset of test cohort with baseline ctDNA sequencing (HR_High vs._ Low=4.96, 95%CI: 2.59–9.51, *P*=1.4×10^−06^; HR_Intermediate vs. Low_=2.05, 95%CI: 1.11–3.81, *P*=0.023; Fig. 3g).

We next focused on patients with tumors harboring *PIK3CA* mutations (n=226), considering the recent approval of inavolisib in combination with CDK4/6i+ET in the first-line setting^33^, with additional ongoing trials investigating PI3K/AKT inhibitors in this therapeutic space (NCT06790693, NCT04862663, NCT07174336). Consistent with prior reports^14,31^, *PIK3CA* mutation status was not associated with PFS (HR=1.02, 95%CI: 0.81–1.28, *P*=0.88). Conversely, CDKPredict yielded robust risk stratification across *PIK3CA* wild-type and mutant tumors (Fig. 3h), identifying a subset of *PIK3CA*-mutant cases achieving durable disease control on CDK4/6i+ET alone (n=85, 37.6%, mPFS=32.9 months, 95%CI: 22.1–not reached), emphasizing the rationale for pursuing risk-adapted therapeutic strategies in this biomarker-selected population. Finally, CDKPredict retained robust risk stratification across clinically relevant subgroups (TFI ≤12 and >12 months, recurrent vs. *de novo* metastatic disease, bone-only disease, presence of liver metastasis, and age) both in the external and MSK-cohort (Extended Data Fig. 6c).

Lastly, we benchmarked CDKPredict against historic risk criteria currently employed for first-line therapeutic escalation in HR+/HER2− MBC, namely TFI ≤12 months. While TFI ≤12 months was associated with inferior outcomes in the external cohort (HR=1.81, 95%CI: 1.41–2.32, *P*=2.8×10^−06^), and TFI high-risk patients (n=149) were largely predicted at high- or intermediate-risk by CDKPredict (Fig. 3i), a critical limitation emerged among patients classified as low-risk (TFI >12 months, n=415). In this population, CDKPredict reclassified a substantial proportion of TFI low-risk patients as high- (n=73, 17.6%) or intermediate-risk (n=151, 36.4%) of early progression, who ultimately achieved short- to mid-term disease control (CDKPredict-high: mPFS=10.1 months, 95%CI: 8.5–13.9; CDKPredict-intermediate: mPFS=14.7 months, 95%CI: 12.4–19.5; Fig. 3i). In an exploratory analysis, we assessed this question in the larger MSK-cohort using out-of-bag risk estimates from the locked ensemble model (Methods). In this analysis, a substantial proportion of TFI low-risk patients were classified as intermediate or high risk by CDKPredict (n=330, 43.9%). Conversely, 16.6% (n=85) of TFI high-risk patients were reclassified as CDKPredict low risk and achieved durable disease control on CDK4/6i+ET (mPFS=31.0 months, 95%CI: 19.3–73.7; Extended Data Fig. 6d). Finally, CDKPredict demonstrated more accurate discrimination at both the 12- and 24-month landmark PFS analyses in the external validation cohort (Fig. 3j), reinforcing its clinical advantage over currently adopted criteria for front-line risk stratification.

### CDKPredict risk classifications reflect clinical and biological plausibility

To assess the clinical and biological plausibility of CDKPredict, we took two complementary approaches. First, we leveraged the natural interpretability of its linear ensemble framework to examine features driving risk predictions. Second, we tested whether risk classifications recapitulated established genomic and transcriptomic programs of CDK4/6i+ET resistance not provided a priori to the model.

For each variable, we quantified the selection frequency across the 100 base learners together with the mean contribution to patient risk-scores (Fig. 4a–b, Extended Data Fig. 7a). Among clinical features, liver metastasis, low estrogen receptor expression (ER≤10%), lack of progesterone receptor expression (PR-negativity), higher number of metastatic organs (≥4), and TFI ≤12 months demonstrated the highest linear increment in CDKPredict risk-scores (+21.1, +18.2, +15.6, +12.3, and +11.8, respectively). Among genomic features, *TP53* LoF (+19.6) and *MDM2* amplification (+15.7) were among the highest-weighted contributors to risk predictions, consistent with prior work establishing their role as major drivers of CDK4/6i+ET resistance through abrogation of DREAM-mediated cell-cycle control^16^. Beyond these, several genomic features prioritized by CDKPredict recapitulated established mechanisms of resistance to CDK4/6i, ET, or both, including *ESR1* mutations^21,46^ (risk-score: +9.9) and *MYC* amplification^20,47^ (+4.4). High TMB (≥10 mutations per megabase) also strongly correlated with risk (risk-score: +10.6; Fig. 4c), consistent with its previously reported association with reduced benefit from CDK4/6i+ET in phase III randomized trials^31^. Conversely, predictions of durable disease control were supported by distinct genomic mechanisms, such as *GATA3* (risk-score: −9.6), which is known to promote luminal differentiation and endocrine dependency in breast cancer^48,49^.

In addition, we quantified feature importance using Shapley additive explanations (SHAP), which decompose each individual prediction into additive feature-level contributions, enabling assessment of population-level importance from the mean SHAP contribution across patients (Fig. 4b). A set of highly prevalent clinical and genomic features consistently carried the highest population-level impact (top four by SHAP: *TP53* LoF: 0.23, PR-negativity: 0.22, liver metastasis: 0.21, and TFI ≤12 months: 0.17). In contrast, low-prevalence alterations with well-established mechanistic roles in CDK4/6i+ET resistance such as *RB1*^11,13,14,29,32^, *BRCA2*^11^, and *BRCA1*^11,50,51^ demonstrated a modest population-level importance (SHAP: 0.02, 0.04, 0.01, respectively), yet conferred large per-patient risk-scores (*RB1*: +15.7, *BRCA2*: +14.6, *BRCA1*: +8.3), highlighting CDKPredict’s ability to preserve the influence of rare but mechanistically validated drivers of resistance.

Beyond features directly implemented in CDKPredict, risk scores demonstrated a significant correlation with broader markers of genomic instability among tumors carrying a sufficient number of copy-number alterations (n=1,085, 85.8%; Methods, Fig. 4d), including fraction of genome altered (FGA; Spearman’s rho=0.27, *P*=6.3×10^−20^; Fig. 4e, Extended Data Fig. 7b) and whole genome doubling (WGD) events (*P*=7.7×10^−5^ by Pearson’s chi-square test; OR_High vs. Low_=2.28, 95%CI: 1.53–3.40, *P*=2.7×10^−05^ by Fisher’s Exact test; Fig. 4f). Evaluating tumor mutational signatures (Fig. 4g), high-risk tumors featured a higher frequency of HRD (OR_High vs. Low_=2.24, 95%CI: 1.43–3.53, *P*=0.0002; Fig. 4h), consistent with its role in promoting CDK4/6i resistance through acquisition of *RB1* loss and other established resistance mechanisms^11^. High-risk tumors further presented an enrichment of dominant APOBEC mutagenesis (OR_High vs. Low_=1.59, 95%CI: 1.10–2.30, *P*=0.0115), emphasizing its key role in CDK4/6i+ET resistance^30^. The distinct representation of genomic signatures across risk-strata reflected their co-segregation with CDKPredict biomarkers (Extended Data Fig. 7c–d), suggesting that the model inherently captures higher-order genomic alterations through its constituent features. Indeed, augmenting the model with dominant tumor mutational signatures (HRD and APOBEC3), genome-wide copy-number features (WGD and FGA), and hemizygosity in tumor suppressor genes associated with CDK4/6i resistance (i.e., *RB1*, *BRCA1*, *BRCA2*, *TP53*, *LATS2*, *NF1*, *PTEN*)^11^ yielded no discriminative improvements (augmented model AUC[t]=0.74, 95%CI: 0.699–0.811, vs. CDKPredict AUC[t]=0.74, 95%CI: 0.699–0.808; Extended Data Fig. 8, Methods).

Finally, we extended this orthogonal validation to the transcriptomic level, leveraging the subset of patients from the external validation cohort with available baseline RNA sequencing^52^ (n=450, 79.8%; Fig. 4i). CDKPredict low-risk tumors were significantly enriched for PAM50^3^ luminal subtypes (OR_Low vs High_=3.58, 95%CI: 2.01–6.50, *P*=3.6×10^−06^; Fig. 4j), whereas high-risk cases were enriched for basal-like and HER2-enriched tumors (OR_High vs Low_=5.89, 95%CI: 3.08–11.72, *P*=2.6×10^−09^), consistent with established associations between genomic features included in CDKPredict and PAM50 transcriptomic profiles^48^ (Extended Data Fig. 7e, **Supplementary Table 7**). At the gene level, CDKPredict risk scores showed the strongest correlation with progesterone receptor RNA expression (Spearman’s rho=−0.44, *q*=9.0×10^−19^; Fig. 4k, **Supplementary Table 8**), recapitulating its established role as a marker of endocrine sensitivity^53^, and secondarily with *AURKA* among variables included in CDKPredict (Spearman’s rho=0.31, *q*=1.1×10^−08^; Extended Data Fig. 7f, **Supplementary Table 8**), corroborating the role of AURKA overexpression in promoting CDK4/6i+ET resistance, as previously shown^17^.

At the pathway level, gene set variation analysis (GSVA) using the Hallmark collection^54^ revealed an overactivation of estrogen signaling pathways among low-risk tumors (Estrogen response early, ER^early^ *q*=9.5×10^−07^; ER^late^ *q*=0.0137 by Mann-Whitney U) and a downregulation of *KRAS* signaling (*q*=0.0078), among others (Extended Data Fig. 7g). Remarkably, ER^early^ signaling showed the most significant correlation with CDKPredict risk-scores (Spearman’s rho=−0.30, *q*=2.7×10^−09^; Fig. 4l, **Supplementary Table 9**), resulting in a negative gene-set enrichment among high-risk patients (*q*=0.0010; Fig. 4m). Notably, a small subset of high-risk tumors (12.5%, n=15) exhibited overactive estrogen signaling by GMM classification (Fig. 4n, Methods), with a prominent overrepresentation of luminal PAM50 subtypes among these cases (13 of 15; OR=5.41, 95%CI: 1.14–51.73, *P*=0.023). Strikingly, these high-risk/estrogen response-high tumors featured a marked enrichment of *ESR1* mutations compared to high-risk/non-estrogen-high cases (53.0% vs 7.0%, *q*=0.0013 by Fisher’s exact test; Fig. 4o), an association not observed among CDKPredict low- and intermediate-risk tumors (Extended Data Fig. 7h). Although based on a small subset and therefore hypothesis-generating, these findings define a specific subset of high-risk cases in which aberrant estrogen signaling, driven by baseline *ESR1* mutations, might represent the primary mechanism of early progression, providing a biological rationale for prioritizing oral selective estrogen receptor degraders (SERDs) as an ET backbone in this population, an approach currently investigated in *ESR1*-selected and unselected first-line clinical trials (NCT04964934, NCT04711252, NCT04546009).

### Recurrent patterns of actionable alterations to tackle resistance

Building on the genomic variables for risk predictions, we next evaluated the representation of actionable alterations across risk strata (Fig. 5a). Leveraging OncoKB^41^ and the MSK-cohort, the frequency of established actionable alterations (OncoKB levels 1–2) increased gradually across risk classes (low-risk: 55.1%; intermediate-risk: 60.2%; high-risk: 67.0%; Fig. 5b). This occurred despite a progressive decline in the frequency of *PIK3CA* mutations among higher risk-strata, reflecting instead an increase in non-*PIK3CA* level 1–2 alterations (Fig. 5c), with 48.0% of high-risk tumors harboring level 1–2 alterations beyond *PIK3CA* compared with 17.0% of low-risk tumors (Fig. 5d). Among these, besides *PTEN*, with proven clinical utility of AKT inhibitors in endocrine-resistant tumors^55^, *ESR1* mutations represent a predictive biomarker for oral SERDs^56,57^, and *ERBB2*-activating mutations have been shown to promote endocrine resistance^26^, potentially reversible by HER2 tyrosine kinase inhibitors^58,59^. Moreover, higher-risk strata featured a higher frequency of high TMB (16.1% in high-risk vs. 1.8% in low-risk; OR_High vs._ Low=10.58, 95%CI: 4.99–25.09, *P*=1.3×10^−13^), a tumor-agnostic biomarker of immunotherapy efficacy^60–62^.

Beyond level 1–2 alterations, high-risk tumors showed a higher representation of alterations with supportive clinical or preclinical evidence of actionability (OncoKB level 3–4, Fig. 5b), with only 15.8% of high-risk tumors lacking any actionable alterations (vs. 35.3% in low-risk; **Supplementary Table 10**). While the frequency of germline or somatic *BRCA2* alterations was significantly higher in intermediate- and high-risk tumors compared to low-risk cases (**Supplementary Table 11**), *BRCA1* (OR_High vs. Low_=11.59, *q*=0.00052) and *PALB2* (OR_High vs._ Low=7.61, *q*=0.0125) were found almost exclusively among high-risk tumors. Given the established role of PARP inhibitors for somatic or germline *BRCA1/2* and germline *PALB2* alterations^63–65^, and prior work supporting the clinical utility from prioritizing PARP inhibition in this context^11^, these data further support the design of EvoPARBreast-01, a phase III randomized trial comparing front-line PARP inhibition plus ET with standard CDK4/6i+ET in tumors harboring germline or somatic *BRCA1/2* or *PALB2* alterations (NCT06380751).

Finally, *TP53* LoF demonstrated the starkest increase across risk-strata (61.1% in high-risk vs. 4.3% in low-risk; OR_High vs. Low_=34.66, 95%CI: 20.82–59.29, *q*=6.8×10^−69^; Fig. 5a). *MDM2* amplification followed a similar pattern (8.4% in high-risk vs. 1.8% in low-risk; OR_High vs. Low_=5.08, 95%CI: 2.22–12.64, *q*=0.0001; Fig. 5a). Notably, the addition of CDK2 inhibitors to CDK4/6i+ET has been previously shown to overcome CDK4/6i resistance in *TP53*/*MDM2*-altered breast cancer^16^, providing a direct mechanistic rationale for evaluating the use of CDK2-targeted strategies in this high-risk subgroup.

Collectively, these findings highlight the potential value of CDKPredict as a backbone for risk-adapted, biomarker-directed therapeutic strategies, linking each risk stratum to a distinct landscape of actionable alterations with supporting clinical utility to tackle endocrine resistance.

### Development of a scalable model for broad clinical deployment

We finally sought to develop a scalable and pragmatic model, termed CDKPredict-S, that could be utilized globally, both in routine clinical practice and as an enrichment tool for clinical trials (Fig. 6a). Two key principles guided this effort: first, limiting the required input to clinical variables routinely and uniformly collected in clinical practice worldwide, and genomic variables to a minimal, essential gene panel accessible across diverse sequencing platforms; second, ensuring robustness against missing data to maintain model calibration at both the clinical and genomic levels. We therefore developed two versions of CDKPredict-S: a clinical-only model relying exclusively on limited clinicopathologic variables, and a clinicogenomic model that also included a restricted set of genomic biomarkers with established clinical relevance in HR+/HER2− MBC and broad availability across different sequencing panels (*PIK3CA*, *PTEN*, *AKT1*, *BRCA1*, *BRCA2*, *ESR1*, *TP53*, *RB1*). To further maximize scalability, both versions were designed to tolerate missing variables by using the average risk contribution as measured in the development set to mitigate systematic distortions of risk scores (Methods).

Following evaluation in the internal hold-out set (Fig. 6b, **Supplementary Table 12**) and fine-tuning using the full MSK-cohort (Extended Data Fig. 9a–b), CDKPredict-S was evaluated across two independent test cohorts (Fig. 6c). Besides the external nationwide multi-institutional cohort utilized for the clinical validation of CDKPredict, we leveraged 455 patients with HR+/HER2− MBC who received first-line CDK4/6i+ET at a single European institution (IEO-cohort), for which baseline genomic sequencing was available in 21.1% of patients (96 of 455) using a tissue-based panel interrogating 523 cancer-related genes^66^ (12.7%, n=58) or a cfDNA-based 50-gene assay^67^ (8.4%, n=38; **Supplementary Table 13**).

In the pooled external cohort, clinical (AUC[t]=0.68) and clinicogenomic (AUC[t]=0.71) risk-scores of CDKPredict-S demonstrated robust risk-discrimination (Fig. 6d–e, Extended Data Fig. 9c–d, **Supplementary Table 12)**. Across risk-strata, both the clinical model evaluated in 1,019 patients (HR_High vs. Intermediate_=1.99, 95%CI: 1.65–2.41, *P*=1.0×10^−12^; HR_High vs. Low_=3.09, 95%CI: 2.50–3.82, *P*=2.4×10^−25^) and the clinicogenomic model evaluated in 660 patients (HR_High vs. Intermediate_=2.12, 95%CI: 1.68–2.68, *P*=3.0×10^−10^; HR_High vs. Low_=3.60, 95%CI: 2.75–4.72, *P*=1.1×10^−20^) demonstrated robust baseline risk stratification (Fig. 6f–g), confirming the clinical validation of the scalable model. Although its discrimination was modestly lower than that of the full CDKPredict model (AUC[t]=0.74), this reflected a deliberate trade-off, restricting input to a minimal set of widely available features in exchange for broad clinical implementation. Following validation, CDKPredict-S was deployed for academic and non-commercial use at https://cdkpredict.com/.

## DISCUSSION

In the present work, leveraging a large and densely annotated clinicogenomic cohort together with independent multi-institutional datasets, we developed and clinically validated CDKPredict, a clinicogenomic model designed to forecast treatment outcomes on front-line CDK4/6i+ET to inform risk-adapted therapeutic interventions in clinical practice.

Over the past decade, several individual prognostic and predictive biomarkers related to CDK4/6i+ET have been developed; however, an integrated effort to combine them for baseline prognostication had not been undertaken. We now demonstrate that integrating clinical and genomic variables within a single prediction framework outperforms standalone biomarkers for baseline risk stratification. Moreover, we developed an interpretable model matching the performance of state-of-the-art machine-learning architectures, while preserving sensitivity to rare but mechanistically validated drivers of resistance (e.g., *RB1*, *BRCA1/2*, *PALB2*, *PTEN*) that conventional penalized models tend to discard. This represents a consequential property given that several of these drivers are directly targetable.

Prediction tools based on key biologic and prognostic features have played an important role in improving outcomes for breast cancer patients in the early stage, but only recently have they begun to be used in the metastatic setting. In this context, CDKPredict exhibited superior clinical validity compared to the high-risk criteria currently adopted in phase III clinical trials such as INAVO120^33^. Furthermore, a major strength of our framework lies in the aggregation of established biomarkers to potentially inform risk-adapted and biologically informed treatments. Indeed, while single gene or pathway-level alterations including *ESR1* and *PIK3CA/AKT1/PTEN* are intended to inform targeted treatments, CDKPredict captures the combined contribution of multiple clinical and genomic features to prioritize alternative regimens in an individualized and risk-adapted fashion. Consistent with this, we observed that CDKPredict classifications aligned with established genomic and transcriptomic states mechanistically linked to distinctive spectra of CDK4/6i+ET sensitivity. These findings validate its biological plausibility and the capability of such predictions to reflect coherent disease states rather than isolated biomarkers. This performance was preserved across cohorts profiled with diverse tissue- and plasma-based sequencing panels and spanning academic and community practice settings, supporting robustness to real-world platforms and practice heterogeneity.

As anticipated, CDKPredict uncovers significant opportunities for pursuing the prospective evaluation of risk-adapted therapeutic interventions. Our model demonstrated consistent capability to detect tumors that were less likely to derive benefit from standard therapy, thereby identifying a population for which intensified front-line treatments could potentially prevent rapid disease progression. In parallel, higher-risk strata were enriched for clinically actionable alterations, coupling risk stratification to biomarker-matched escalation, such as PARP inhibition for *BRCA1/2* or *PALB2* alterations, PI3K, AKT, and mTOR inhibitors for PI3K/AKT pathway alterations, CDK2 inhibitors for *TP53* LoF or *MDM2* amplification, and oral SERDs for *ESR1*-driven estrogen signaling. Moreover, CDKPredict readily identified a subgroup of patients exhibiting long-term benefits from standard therapy alone, for whom treatment escalation is unlikely to provide additional benefit given the financial and health-related toxicities. In the same context, CDKPredict could potentially address the clinical task of identifying patients with tumors exhibiting pronounced endocrine sensitivity, who could represent an appropriate risk-defined population for de-escalated treatment approaches. Indeed, although comparable benefits from early versus deferred use of CDK4/6i have been observed^68^, the lack of reliable biomarkers represents a key limitation to the confident adoption of such de-escalation strategies in clinical practice.

Beyond these clinical applications, our study highlights the broader potential for such models in varied practice settings. To realize this potential, we additionally developed CDKPredict-S, a scalable version requiring routinely collected clinical variables and a minimal gene panel, which retained robust risk stratification across two large independent cohorts, positioning it as a practical enrichment tool for clinical trials and a decision-support aid in routine practice worldwide. Together, these models are well-suited for implementation in prospective studies investigating biologically informed, precision-based, and risk-adapted treatment interventions.

Our study has limitations. First, while CDKPredict is intended for risk stratification in patients receiving CDK4/6i+ET, the absence of a non-CDK4/6i comparator arm precludes definitive conclusions about treatment-specific effects. Nevertheless, the biological plausibility of genomic and transcriptomic features, many of which have been previously implicated specifically in CDK4/6i+ET resistance and with established clinical utility in phase III clinical trials, supports our hypotheses and conclusions. Second, while our clinicogenomic framework proposes baseline risk stratification for CDK4/6i+ET, its value in informing alternative therapeutic strategies to improve clinical outcomes has not been addressed in the present study.

In conclusion, we present the validation of a translational framework capturing the multidimensional biology underlying distinct benefits from CDK4/6i+ET. In an era of increasingly complex and biologically driven therapeutic strategies in HR+/HER2− MBC, we provided a foundation for the prospective evaluation of risk-adapted, precision-based therapeutic interventions.

## ONLINE METHODS

### Study cohort and prospective sequencing

The Memorial Sloan Kettering (MSK) Breast Translational cohort encompassed 1,264 individuals with HR+/HER2− breast cancer who received routine care at MSK, with prospectively collected and manually curated clinical data. Inclusion criteria consisted in the receipt of first-line CDK4/6i+ET, together with baseline tumor genomic sequencing, defined as sequencing performed on a tumor sample collected either before or within 28 days from treatment start. Additional criterion for study eligibility included the receipt of first-line CDK4/6i+ET for at least 28 days. Patients that received investigational agents together with CDK4/6i+ET or induction chemotherapy before CDK4/6i+ET were excluded from the study population. For patients with multiple samples satisfying the temporal sequencing criteria, a manual review of the cases was conducted, prioritizing samples collected at a more recent time relative to the start of CDK4/6i+ET, utilizing the tumor cellularity and sequencing depth as a second criterion for samples selection. The present study was approved by the Memorial Sloan Kettering Cancer Center (MSK) Institutional Review Board (IRB) and all patients provided written informed consent for tumor and paired normal DNA sequencing and review of medical records for clinical annotations.

The external, nationwide multi-institutional cohort encompassed 564 individuals affected by HR+/HER2− breast cancer, who received first-line CDK4/6i+ET in community or academic settings, with available patient-level clinicogenomic data resulting from referral to a tertiary-level sequencing facility for baseline tumor genomic profiling on samples collected before and up to 28 days from starting first-line CDK4/6i+ET. The same exclusion criteria, including a treatment duration below 28 days, investigational agents or induction chemotherapies among others were utilized for the study eligibility in the external multi-institutional cohort.

For validating a scalable model for variable-resource settings, in addition to the abovementioned external cohort, we leveraged a cohort of 455 patients, who received routine care in a single-center institution in Italy (European Institute of Oncology, *IEO-cohort*). Inclusion criteria included HR+/HER2− MBC and receipt of first-line CDK4/6i+ET for at least 28 days. Patients with or without baseline genomic sequencing (performed before or within 3 months from start of first-line CDK4/6i+ET) were included in the study cohort. The temporal criterion for tumor sequencing was expanded relative to the MSK-cohort to facilitate the collection of a larger external independent validation cohort, hypothesizing that secondary resistance mechanisms would be less likely to emerge only after a limited duration of treatment with CDK4/6i+ET.

### Genomic sequencing

For patients included in the MSK-cohort, samples were sequenced in a CLIA-certified laboratory using the FDA-cleared, tumor-normal MSK-IMPACT sequencing panel, as described previously^34^. Patients provided consent for tumor profiling under an institutional IRB-approved research prospective protocol, ‘Tumor Genomic Profiling in Patients Evaluated for Targeted Cancer Therapy’ (NCT01775072). Tumor type and purity were assessed by a dedicated pathologist from hematoxylin and eosin-stained slides of tumor samples. In detail, DNA was extracted from formalin-fixed, paraffin-embedded tumor and matched normal (peripheral blood) samples. Targeted sequencing was conducted with custom DNA probes against exonic and intronic regions according to the MSK-IMPACT version, encompassing 341 genes (n = 4 samples, 0.3%), 410 genes (n = 127, 10%), 468 genes (n = 666, 52.7%) and 505 genes (n = 467, 36.9%). Tumor samples with no detectable somatic alterations likely due to low tumor content or low sequencing depth (<100x) were excluded (n=30). Tumor mutational burden (TMB) was calculated as the number of non-synonymous mutations per megabase sequenced (mutations per megabase, Mut/Mb). TMB high (TMB-H) was defined as ≥10 Mut/Mb, consistent with the FDA-approved indication for the agnostic use of immune-checkpoint inhibitors, as previously defined^60^.

Somatic genomic alterations were filtered for ‘oncogenic’ or ‘likely oncogenic’ alterations using OncoKB, a precision oncology knowledge-base that tracks the effects of cancer variants and their potential clinical actionability (http://oncokb.org). Genomic alterations were classified as actionable using a level of evidence from 1 to 4, where Level 1–2 alterations indicate a standard therapeutic intervention and levels 3A-4 including investigational therapeutic alterations, which may direct a patient toward a clinical trial relevant to that biomarker. Germline alterations were included only for *BRCA*1/2 and *PALB2*, in line with their previous relevant prognostic role for patients receiving CDK4/6i+ET.

Allele specific copy number, tumor purity and ploidy were inferred using FACETS (v0.5.14)^69^. Copy number quality call was evaluated using a set of consecutive FACETS quality control metrics (https://github.com/taylor-lab/facets-preview/). A total of 1,085 tumors (85.8%) passed these filters and were included in the analyses of fraction genome altered (FGA) and whole-genome doubling events (WGD). FGA was defined as the proportion of the profiled genome with absolute log_2_ copy number changes greater than 0.2. WGD was inferred as previously described^70^, with tumors classified as WGD-positive when more than 50% of the autosomal genome exhibited a major copy number equal or greater than 2.

Genomic signature analysis was conducted using Signature multivariate analysis (SigMA; https://github.com/parklab/SigMA), whose validation for imputing HRD and APOBEC leveraging the MSK-IMPACT sequencing assay has been previously validated^30,71^. As previously defined, only samples with at least n=5 SNVs were considered for mutational signature prediction (n=946, 74.8%). To increase the sensitivity of the analysis and to gauge a larger spectrum of genome-wide mutagenic processes extending beyond covered regions, both silent mutations and SNVs originating from off-target reads were included, using as a quality filter a sequencing depth of at least ≥100 reads in the involved genomic region, together with at least 5% reads supporting the alternate variant (variant allele frequency of 5%).

In the external multi-institutional cohort, tumor sequencing was performed using a commercially available genomic sequencing panel interrogating up to 648 cancer-related genes, as previously validated^43^. In detail, genomic profiling was conducted using the solid tumor Tempus platforms xT and the legacy xO assay, while plasma-based profiling was conducted using the liquid biopsy xF platform^45^. Among 564 patients, 417 (73.9%) underwent tissue-based testing and 147 (26.1%) underwent plasma-based testing. The specific assay distribution was xT (n=6), xT.v2 (n=62), xT.v3 (n=81), xT.v4 (n=267), xF.v2 (n=146), xF.v3 (n=1), and xO (n=1).

For patients included in the IEO-cohort, baseline genomic sequencing was available for 21.1% cases (96 of 455). In detail, tumor genomic sequencing was conducted using an analytically validated and commercially available hybrid capture, tumor-only solid-tissue sequencing panel interrogating 523 cancer-related genes^66^ for n=58 cases (60.4%). For the remaining 38 cases (39.6%), tumor sequencing was conducted using an analytically validated and commercially available cfDNA-based tumor-only sequencing assay targeting 50 cancer-related genes^67^.

### Clinical data annotation for the MSK-cohort

Clinical data was gathered from the MSK Breast Translational Program’s clinical database, which contains comprehensive, longitudinal information on patient demographics, pathology, treatment regimens, and clinical outcomes. Structured data was systematically and manually curated using a standardized clinical data annotation process by a dedicated team of clinical data annotators and were maintained under rigorous quality-control procedures by experienced data managers. The data lock for the analysis was set on October 29, 2025.

Progression-free survival was defined as i) a clinical or radiographic disease progression prompting change in systemic therapy or recommendation for ablative local therapy directed at site(s), or ii) any cause of death. Patients lost to follow-up, or who were still receiving first-line CDK4/6i+ET at last follow up, or that discontinued treatment without evidence of either clinical or radiological progression (e.g. toxicity, patient preference or insurance coverage) were right censored. When endocrine therapy or CDK4/6i changed to another compound (endocrine compounds or CDK4/6i, respectively) for reasons other than disease progression (e.g. toxicity, patient preference or insurance coverage), the time on successive regimens was combined to more accurately capture the real-world PFS. When patients discontinued CDK4/6i after 28 days due to toxicity while continuing the endocrine agent alone, the overall PFS achieved before progression or death on the same regimen or up to the right censoring time were combined.

### Train and test stratification in the MSK-cohort

For developing the model, the MSK-cohort was divided into a training set (70%, n=884) and an internal validation set (30%, n=380). The train-test split was conducted in a stratified manner, balancing the rate of i) events, ii) liver metastasis, iii) treatment-free interval ≤12 months, and iv) the availability of FACETS (passing quality control criteria, as abovementioned) between the train and test set. The abovementioned factors were selected to ensure a similar median-follow up (median follow up by reverse Kaplan Meier method in the train set of 49.7 months, 95%CI 26.8–79.9; in test set of 50.6 months, 95%CI 24.9–74.3), a balanced representation of relevant prognostic clinical features for preliminary model validation, and to achieve a balanced representation of FACETS-level signatures for assessing the clinicogenomic model augmented with higher-order genomic features.

For evaluating the balanced representation of clinical and genomic variables between the train and test set, univariable tests were conducted using the Fisher’s exact test (categorical variables). Furthermore, for evaluating the genomic representation of features between the train and test cohort, we leveraged an adversarial random forest classifier encompassing n=100 trees, which was trained to distinguish instances belonging to the training set or test set using genomic features present in at least 1.5% of the overall MSK-cohort. The evaluation was performed on hold-out samples using a 10-fold cross-validation. The model discriminative performance was measured with the area under the receiver operating characteristic curve (AUC), with a score equal to 0.5 representing a features distribution not reflecting an imbalanced split of genomic variables in a higher-order dimension between the train and the internal validation set. In addition, for evaluating the multi-dimensional embedding of genomic features, a principal component analysis (PCA) was conducted (extracting the first-two principal components). To quantify the contribution of original features to the two first principal components in the representative illustration, PCA loadings were extracted from eigenvectors accessed using the pca.components_ attribute in scikit-learn.

### Features engineering

Clinical and genomic data, collected as abovementioned, were utilized for developing a machine learning framework designed to predict the risk of early progression from first-line CDK4/6i+ET.

Specifically, for clinical variables, features selection was conducted using a knowledge-based criteria that included clinical variables relevant to outcomes with CDK4/6i+ET. Continuous variables were engineered to result in a categorical representation to maximize the clinical interpretability of the model, broad applicability and to mitigate differences originated from inter-observer or pre-analytical factors (e.g., percentage of nuclei showing estrogen and progesterone receptors staining). The final set of features included in the model comprised: presence or absence of metastasis involving the liver (i) or central nervous system (ii) at the time of CDK4/6i+ET initiation; iii) presence of bone-only disease; iv) treatment-free interval equal or below 12 months, defined as tumor recurrence occurring during endocrine therapy or within 12 months from the end of adjuvant therapy for either toxicity or treatment plan termination; v) high-grade tumor as per Nottingham Histologic Score^72^; for patients with multiple samples or without available metastatic samples, those collected closer to the start of first-line CDK4/6i+ET and samples from metastatic sites were prioritized. vi) de novo metastatic disease, defined as a newly diagnosed metastatic disease or with locoregional extension considered not amenable for curative-intent locoregional treatments. vii) age at metastatic diagnosis under 50 years; viii) use of fulvestrant as an endocrine backbone; for patients that as per physician’s choice were switched from aromatase inhibitors to fulvestrant without evidence of clinical or radiological progression, or according to the emergence of molecular biomarkers not present at baseline (e.g. novel detection of an *ESR1* mutation) were considered as receiving fulvestrant; ix) negative progesterone receptor, defined as 0% of tumor cellular nuclei showing receptor staining; for patients with multiple samples, priority was given to the sample collected at a more recent time relative to first-line treatment start and to those collected from metastatic sites; x) estrogen receptor percentage equal or below 10%; and xi) above 10% and equal or below 80%, using the ≥80% as a reference category to reduce collinearity in the variables; xii) number of distant metastatic organs equal to 2 or 3 or xiii) equal or above to 4, using as reference the presence of at maximum one distant metastatic organ or locoregional disease to reduce collinearity; xiv) genomic sequencing conducted on a metastatic sample.

For selecting genomic variables to be included in the model, we considered somatic tumor variants annotated as oncogenic or likely oncogenic by OncoKB^40,41^. For considering differences in the biology of distinct alterations classes, which is particularly relevant in the context of breast cancer, alterations affecting oncogenes were classified as [Gene_MUT_] for genomic mutations including single nucleotide variants and small insertion-deletions (indels), [Gene_AMP_] for amplifications, and [Gene_FUS_] for gene fusions or structural variants. Assuming a common role of pathogenic variants to converge on a loss of function of tumor suppressor genes, oncogenic and likely oncogenic alterations belonging to distinct mutational classes were considered together for tumor suppressor genes as [Gene_ALT_]. Tumor suppressor genes were defined according to OncoKB (https://www.oncokb.org/cancer-genes). Amplifications including *CCND1*, *FGF4*, *FGF3*, and *FGF19* were merged in 11q13 amplicon considering their co-localization within the same genomic amplicon and to avoid unstable models estimates due to collinearity. In the same manner, deletions involving *CDKN2A* and *CDKN2B*, whose co-deletion represent a recurrent genomic event in breast cancer, were classified together as amplicon 9p21_DEL_. For selecting genomic features to include in the model, we employed a frequency-based features selection to include genomic alterations present in at least 1.5% samples to avoid possible statistical noise derived from rare features, resulting in n=42 genomic variables. Besides them, according to a biology-based priori, we included *BRCA1* (n=16), *PALB2* (n=11), *FAT1* (n=16), and *PIK3R1* (n=16) alterations, whose prognostic importance has been widely established in breast cancer by our group among others^11,14,50,51^. To account for previously reported technical-related differences across sequencing platforms^73^, rather than being normalized and engineered as a continuous variable, the TMB score was one-hot encoded considering the absolute number of mutations, including i) 6–9 Mut/Mb (n=153, 12.1%) and ii) ≥10 Mut/Mb (n=95, 7.5%), using the TMB: 0–5 Mut/Mb as the reference (n=1,016, 80.4%) to reduce collinearity. Collectively, 48 genomic features were included in the genomic and clinicogenomic model.

For developing the augmented model leveraging higher-order genomic variables to benchmark with CDKPredict, besides the abovementioned variables, we included tumor mutational signatures, FGA, and hemizygosity in tumor suppressor genes, this latter defined from the allelic-specific copy number analysis (using FACETS) as a total copy number of n=1 together with a lesser copy number of n=0 involving the specific gene. Specifically, tumor mutational signatures derived with SigMA were categorized to represent APOBEC- and HRD-dominant groups (by one-hot encoding), using other signatures as the reference level. For categorizing FGA, we utilized the Youden’s J statistic to define the optimal cutoff maximizing the specificity and sensitivity to detect patients experiencing a PFS event within 12 months, while excluding samples that were right censored before 12 months. The 12-month timeframe was selected to emphasize the prediction discrimination in a time-frame useful for employing baseline, risk-informed decision making in patients with breast cancer considered for first-line therapy. The FGA cutoff was determined at a 0.35 threshold, as calculated in the training set and applied for classifying samples in the internal validation set. For selecting genes showing hemizygosity, we performed a univariable Cox model for each tumor suppressor gene correcting the analysis for fraction of the genome carrying loss of heterozygosity events (fraction loss of heterozygosity), as determined with FACETS, and WGD events, with the correction conducted to isolate the prognostic role of hemizygosity events from co-occurrent passenger events in the context of large scale genomic instability. Ultimately, tumor suppressor genes showing a false discovery rate <0.1 (calculated with the Benjamini-Hochberg procedure) and with a prior demonstrated role to promote endocrine resistance were selected, ultimately including hemizygosity events in *RB1*, *BRCA1*, *BRCA2*, *TP53*, *LATS2*, *NF1* and *PTEN*.

### Rationale for developing CDKPredict

For developing CDKPredict, we developed a supervised time-to-event machine learning framework which integrates structured data to predict time-dependent outcomes, available at https://github.com/lboscolobielo/CDKPredict.

In detail, CDKPredict was specifically designed to address a main limitation of conventional feature selection and modeling when applied to sparse clinicogenomic datasets, namely the tendency to remove or scarcely leverage infrequent yet meaningful variables. This is relevant with structured genomic data, in which genomic features are characteristically sparse, i.e., a single genomic alteration might be represented in a minority of cases, and biologically informative events could therefore exhibit a limited prevalence. This has been particularly and widely demonstrated in breast cancer, wherein rare yet crucial genomic alterations yielding resistance to CDK4/6i+ET may occur in a restricted number of cases. Under these assumptions, Cox-penalized survival models may shrink the coefficients of rare features toward zero unless their contribution to the objective function is large enough to surpass the regularization term. This might not occur for rare yet biologically meaningful features, particularly when co-occurring with other relevant and more prevalent variables in the overall training cohort to contribute to maximizing the objective function:

$$\hat{\beta }=\text{arg}\;\underset{\beta }{\text{max}}\left(\ell \left(\beta \right)-\lambda \sum_{k=1}^{p}\left|\beta_{k}\right|\right)$$

Where $\hat{\beta }$ represents the calculated vector of model coefficients, $\ell \left(\beta \right)$ notes the Cox partial log likelihood to be maximized by the features vector, λ the regularization parameter of the Cox model, and $\sum_{k=1}^{p}\left|\beta_{k}\right|$ the L1 penalty for Least Absolute Shrinkage and Selection Operator (Lasso) models. Altogether, this might limit the ability of the prediction framework to capture low frequency but prognostically relevant variables, which have been extensively demonstrated to have a critical role in determining resistance to CDK4/6i+ET in breast cancer.

In addition, to facilitate the potential clinical implementation of the model in routine care, we aimed to establish a model with clear interpretability, leveraging therefore a Cox-model framework.

### CDKPredict framework

For developing CDKPredict, we developed an ensemble machine learning framework encompassing n=100 penalized Cox-models as base learners, building on a previously developed framework developed at MSK^74^, to result in a single-patient risk score prediction. In detail, each base learner consists in a L1-penalized Cox proportional hazard model, wherein each single model was fitted on a distinct subset of the training set defined with a resampling with replacement strategy (bootstrap). Such approach was deliberately employed to increases the heterogeneity in the features representation across base learners, subjecting each single model to a distinct patient-level and features-level representation of the overall training set. Similarly, the L1-penalty term was determined to emphasize the relative importance of potentially rare yet meaningful features by forcing each base learner to maximize the objective function leveraging a distinct subset of features.

In addition, cognizant on the intrinsic tendency of the L1-penalty to over shrink the relative importance of infrequent yet potentially important variables, we leveraged a previously developed framework^39^ to employ a feature-specific penalty term across each distinct base learner (i.e., adaptive lasso). In detail, each of the n=100 base learners was fitted using feature-specific penalty factors estimated from a prior L2-penalized Cox model fitted on the specific bootstrap subcohort of each base learner. Herein, the regularization strength of the L2-penalized model was selected using the best 3-fold cross validation score, considering a hyperparameter space included in the log-uniform scale between −2 and 1. The score was defined as the mean time-dependent AUC utilizing n=10-time bins from 3 to 36 months. Such time frame was selected to optimize the model for discriminative accuracy in a clinically meaningful timeframe for potential clinical decision-making in breast cancers considered for first-line CDK4/6i+ET. Each feature-specific penalty term for the subsequent adaptive lasso optimization was determined as the inverse of the absolute value of the L2-penalized coefficients. Collectively, variables with larger initial coefficients, therefore more important for increasing the partial log-likelihood of the L2-penalized Cox model, received smaller penalties in the subsequent adaptive lasso step, whereas variables with negligible effects were penalized more strongly.

Finally, each L1-penalized base learner was fitted utilizing variable-specific penalty weights, calculated as above, on the respective bootstrap-resampled cohort. In detail, for finding the optimal regularization strength of the L1 penalty term, the initial set of candidate penalty terms was defined by fitting the L1-penalized model to the bootstrap-resampled cohort, gathering a set of n=100 coefficients ranging from a maximum regularization strength setting all coefficients to 0, until a minimal penalty not performing any feature selection (i.e., setting the coefficient of a feature to 0). Subsequently, the optimal regularization strength was selected according to the best time-dependent AUC score, with the same definition as above and calculated within a 3-fold crossvalidation framework.

### Calculation of CDKPredict risk-scores

During each base learner training, a risk-score was computed on out-of-bag samples. Critically, such strategy was employed to gather an objective and reliable assessment of the model performances on the overall cohort employed for training the ensemble model. Indeed, such strategy allowed us to mitigate the risk of data leakage and overconfident predictions of the model resulting from risk-scores computed on samples utilized for model training, enabling a reliable estimation and calibration of the model during its early development stage. Moreover, we assumed an objective estimation of train scores would further enable the definition of reliable cutoffs which could potentially better generalize once considering a single-patient prediction of independent samples. For each patient, an individualized continuous score was defined from the mean of the predicted risk scores across base learners. Finally, risk scores were min-max normalized between 0 and 100 using the 1^st^ and 99^th^ quantiles of raw scores to avoid distributional shifts from relevant outliers.

For predictions on samples not utilized during model fit, risk scores were defined by leveraging the full set of n=100 base learners, with each computing the continuous scores derived from the vector multiplication encompassing the sample-specific features and the base-learner specific coefficients.

### Definition of discrete risk groups by CDKPredict

Following the definition of patient-specific risk scores, we next aimed to gather a risk-class definition to facilitate the clinical implementation of model predictions for informing potential decision making. For this scope, we employed a Gaussian mixture model (GMM) using the training risk scores measured as abovementioned to result in a data driven classification. Specifically, patient-specific training risk scores were modelled using pre-defined Gaussian components (n=2 for the clinical and genomic only model, n=3 for the clinicogenomic model, resulting from the concordance analysis as defined in the study results) to fit a GMM. After fitting, the component specific means, variances and weights (i.e., proportion of samples included in each component) were extracted. Finally, risk-group thresholds were defined by calculating the root (i.e., value equal to 0) of the difference between the adjacent components’ probability density functions, corresponding to the point at which their densities were equal and thus where components intersected, which was calculated using Brent’s method as implemented in scipy.optimize.brentq.

Once estimated in the training cohort, the cutoffs were locked and subsequently applied unchanged for risk-group assignment both in the training set and in the validation sets. Importantly, this approach enabled the definition of risk groups in a data driven manner, without leveraging arbitrary and possibly not biologically reasonable quantile-based cutoffs.

### Development of CDKPredict and validation strategy

In the preliminary development of CDKPredict, the ensemble model was trained using the abovementioned framework on the MSK-cohort training set, encompassing 70% (n=884) of the MSK-cohort cases. For the internal validation of CDKPredict, risk-scores and the corresponding risk classes were defined on independent samples encompassed in the internal validation cohort (30% of the development cohort, n=380).

For gathering the external validation of CDKPredict, before deployment, the model was refitted and risk-cutoffs between risk-groups fine-tuned using the full MSK-cohort (n=1,264 cases), as per standard machine learning practice. This strategy was deliberately employed for optimizing the ensemble model to the full set of variables and observations in the MSK-cohort, wherein rare yet meaningful features could have been allocated in an unbalanced manner between the train and the internal validation set. After model training on the full cohort, the final model coefficients across the n=100 base learners together with the fine-tuned cutoffs were locked and applied without any change to the external cohorts. For each patient, the complete preprocessing and feature-encoding pipeline used during training was replicated, after which the locked vectors coefficient were used to generate continuous risk scores and the corresponding risk-group assignment.

### Machine learning and deep-learning architectures utilized for benchmarking CDKPredict

For benchmarking CDKPredict to currently available gold-standard machine learning architectures, we employed a series of machine-learning and deep-learning survival models accommodating right-censored data.

First, we aimed to benchmark CDKPredict to neural networks, building on the hypothesis of neural layers to potentially capture hidden but meaningful interactions occurring between genomic and clinicogenomic variables. For developing deep-learning networks optimized for right-censored data, we leveraged a previously developed framework^75^, consisting in a PyTorch-implemented neural architecture using the Cox partial likelihood as the objective function. For developing a multilayer perceptron (MLP), we encoded a fully connected feed-forward neural network which was coupled to a Cox proportional hazard output layer to compute patient-specific risk scores. Furthermore, we designed a residual neural network building on a linear path behaving similarly to a standard Cox model, while leveraging a parallel fully connected network to possibly capture feature interactions occurring between clinicogenomic features. Collectively, the model was composed of a bias-free linear path, connecting the input matrix together with features-specific coefficients to a one-dimensional output neuron, with a second path consisting in a MLP, with the two paths converging to a final patient-specific risk score, with the latter calculated by the sum between the linear path and the interaction path.

Furthermore, we leveraged state-of-the-art machine learning models for tabular data, namely a survival random forest, and eXtreme Gradient Boosting (XGBoost) model using a Cox survival objective (utilizing the option objective=“survival:Cox” from https://github.com/dmlc/xgboost) for managing right-censored data.

For further comparing an ensemble machine learning framework, we replicated a model which was previously validated to predict time-dependent outcomes from immune-checkpoint inhibitors in a pancancer cohort at MSK and among other institutions^42^, namely SCORPIO. In detail, we employed an ensemble prediction model resulting from a survival random forest, a non-kernelized support vector machine, and a ridge-penalized Cox regression. For computing risk scores, a sum of linear scores resulting from the three models was obtained following a min-max normalization (from 0 to 100) to enable a common scale, as conducted in the original implementation and validation of the model.

### Optimization of the comparator models

For optimizing the benchmarking models, we leveraged the training cohort to employ Optuna (https://github.com/optuna/optuna), a Bayesian-based hyperparameter tuning framework wherein the hyperparameter search was driven by the C-index metric.

Within each Optuna trial, hyperparameter optimization was conducted using a 5-fold cross-validation, which was stratified for balanced splits considering PFS events to ensure a comparable right-censored distribution between the different folds. Moreover, the stratified K-fold cross validation was undertaken to mitigate the risk of optimizing the model according to a single validation cohort potentially under-representative of the overall population, which could particularly be true for sparse and heterogeneous biological data. Parameters were ultimately selected according to the maximum C-index across folds utilizing n=500 Optuna runs.

For neural networks, considering the cohort size and the modest dimension of the features vector, we investigated shallow neural network architectures to prevent model overfitting. In detail, for MLP, the neural network depth was treated as a hyperparameter, with one or two hidden layers explored. The number of neurons embedded in each hidden layer was sampled from a discrete set (128, 64, 32, 16 nodes). The in-between layers nonlinear activation function was treated as a categorical hyperparameter between rectified linear units (ReLU), exponential linear units (ELU) and leaky rectified linear units (LeakyReLU). Dropout regularization was selected as a hyperparameter ranging from 5% to 40%. Considering the categorical representation of features, batch normalization between hidden layers was disabled. Weights were initialized using Kaiming uniform initialization, with the nonlinearity argument adapted to the selected activation; since Kaiming initialization in PyTorch directly supports relu and leaky_relu, ELU networks were initialized using the leaky_relu setting as an approximation. The AdamW optimizer was used to optimize both learning rate and the decoupled weight decay. In detail, the learning rate was sampled log-uniformly from 1×10^−6^ to 3×10^−3^, while the weight decay parameter was sampled between 1×10^−6^ to 1×10^−1^. The number of epochs was likewise set as a hyperparameter (from 200 to 2000), to simulate the model evaluation in the MSK validation set wherein the use of the validation cohort for early stopping during model training would have resulted in data leakage and overconfident estimates. A batch size corresponding to ¼ of the training cohort was used to prevent the model from converging to poor/sharp local optima, albeit using a significant number of samples for each backward propagation to maintain a representative approximation of the overall cohort during optimization. The same optimization scheme and strategy was employed for the residual neural network.

To optimize the random forest survival model, hyperparameters included the number of trees (from 100 to 1,000), maximum depth (from 4 to 9), minimum number of samples for split (from 2 to 20), minimum number of samples for a terminal node (from 5 to 50).

For XGBoost, hyperparameters included tree depth (4 to 9), row and column subsampling for each tree (from 0.5 to 1.0), L1 regularization (log-uniformly sampled between 10^−8^ and 10), L2 regularization (log-uniformly sampled between 10^−8^ and 10), minimum child weight (log-uniformly sampled between 10^−8^ and 100) and max_delta_step from 0.1 to 10. For each Optuna run the number of boosting rounds was set at 1,000.

For the ensemble model architecture, we adopted the same hyperparameter tuning strategy as previously described^42^. Briefly, a grid search was employed using a 5-fold cross validation scheme. Hyperparameters included in the grid search included for the random forest: number of estimators (from 100 to 1100, with steps of 100), maximum tree depth (from 2 to 22, with steps of 2), minimum number of samples for terminal nodes (from 2 to 22, with steps of 2), and minimum samples for proceeding with tree splits (from 2 to 22, with steps of 2); for the support vector machine: alpha penalty (from 2^−12^ to 2^13^, with exponential steps of 2); for ridge regression: regularization strength (with a uniform sampling from 10^−5^ to 10^5^), as utilized in the original implementation of the model.

### Generation of risk scores by comparator models and benchmarking to CDKPredict

To generate risk scores, each model was refitted on the overall training set using the set of hyperparameters as defined in the abovementioned optimization step. Thereafter, the internal validation set was employed to perform single-sample predictions. For each specific model, risk scores from the validation samples were normalized using a 1^st^ and 99^th^ quantile computed on the training set.

For the benchmarking analysis, continuous risk scores were employed to calculate the C-index and time dependent AUC. In detail, the time dependent AUC was calculated as the mean across a time range between 6 months and 60 months using 10-time bins. In addition, n=1,000 bootstrap reiterations were computed for calculating the 95% confidence interval across different representations of the internal validation set, using the same original model fitted on the training set. Similarly, n=1,000 bootstrap reiterations were computed for assessing the C- index between the models. The use of the internal validation set instead of the training set was selected due to the Optuna optimization process, which would have resulted in overconfident estimates for the competing models using predictions obtained on the same samples employed for hyperparameter tuning. Besides the C-index and time dependent AUC, for assessing the stability of model predictions to dataset shifts, which represents a critical attribute for independent single-sample predictions relative to the risk-classes cutoffs determined on the training set, we compared the distribution of normalized risk scores between the training set and internal validation set. Since CDKPredict inherently generates out-of-bag predictions, an analogous procedure was implemented for all competing models by deriving risk scores from out-of-fold predictions within a 10-fold cross-validation framework in the training set prior to final model fitting. In detail, the Wasserstein distance was used to measure the distributional shift between the training and the internal validation risk distributions, with lower values representing higher distributional stability. This assumption and analysis were undertaken considering the similar distribution of genomic and clinical variables and the overlapping clinical outcomes between the two sets. The Kolmogorov–Smirnov test was instead employed to test whether the cumulative distributions of the two sets were significantly different.

### Development of CDKPredict-S for baseline risk stratification in variable resource settings

Cognizant on the fact genomic sequencing remains unavailable in many clinical settings or is represented by panels interrogating a restricted number of genes, we intended to develop a scalable model, namely CDKPredict-S. Specifically, CDKPredict-S was designed to conduct a reliable risk-predictions irrespective of the receipt of baseline somatic genomic sequencing, degree of missing variables, and to rely on a restricted number of variables to facilitate its large-scale implementation.

For patients receiving baseline genomic sequencing, we developed a clinicogenomic version of CDKPredict-S designed to employ clinicogenomic variables for risk predictions. Specifically, CDKPredict-S was developed including the same clinical variables of CDKPredict; for genomic variables, CDKPredict-S was built using a restricted number of genomic biomarkers with established clinical relevance in HR+/HER2− MBC, that is having an established prognostic/predictive role for first-line CDK4/6i+ET, or representing actionable biomarkers for targeted therapies and currently investigated in clinical trials in the first-line setting for HR+/HER2− MBC; the final set of genomic variables for the scalable model included *PIK3CA*, *PTEN*, *AKT1*, *BRCA1*, *BRCA2*, *ESR1*, *TP53*, *RB1*. Thereafter, CDKPredict-S was developed using the same framework and risk-group definition as CDKPredict. For patients not receiving genomic sequencing, we developed a second version of CDKPredict-S, which was designed to rely on the clinical risk-estimation for computing risk-predictions. In detail, a second CDKPredict-S model was trained and validated using the abovementioned clinical variables, together with clinical-only cutoffs to define cases at high-, intermediate- and low-risk of early progression to CDK4/6i+ET.

To mitigate systematic risk distortions originating from missing variables, CDKPredict-S was further designed to learn the overall prevalence of features from the development set (MSKcohort) to estimate the marginal risk-contribution; such decision was undertaken hypothesizing the prevalence of the detected clinicogenomic features among 1,264 cases could approximate the prevalence of the variable among the general population of patients with breast cancer candidate for front-line CDK4/6i+ET. For estimating the baseline marginal risk-contribution of the feature, the overall mean coefficients originating from the n=100 base learners of the final model refitted on the overall MSK-cohort before deployment were multiplied for the learned prevalence of the features, resulting in a baseline risk-score for the missing variable and calibration of the model in risk estimation relative to the risk-strata cutoffs. Importantly, CDKPredict-S was designed to rely on the marginal risk-contribution from the development set rather than conducting data imputation considering the lack of biological and clinical plausibility in estimating a missing categorical feature from the specific variables included in CDKPredict-S.

### Transcriptomic analysis

The transcriptomic analysis was conducted on a subset of cases included in the external cohort with baseline whole-exome transcriptome sequencing (n=450 of 564, 79.8%). Samples were processed using the Tempus RS RNA sequencing assay. Briefly, exome capture was performed using IDT xGen Exome Research Panel probes on a minimum of 50ng of total RNA extracted from FFPE tissue. Libraries were sequenced on the Illumina HiSeq 4000 platform to a target depth of 30–50 million reads. The bioinformatics pipeline utilized Kallisto (v0.44.0) for transcript-level pseudo-alignment and quantification against the Ensembl GRCh37 (release 75) reference. Gene-level counts were derived by summing the abundance of protein-coding transcripts covered by the RS panel.

For the downstream RNA-seq analysis, first, low-expressed genes across the study cohort were removed to retain genes totaling at least 1 count per million across at least 10% of the cohort (passing from 18,890 to 18,208 genes). Next, raw read counts were normalized using the variance stabilizing transformation (VST) as implemented in DESeq2 using the PyDESeq2 package (https://github.com/scverse/PyDESeq2).

For computing PAM50 intrinsic subtypes, we utilized the genefu package (https://github.com/bhklab/genefu), as previously validated^3^, to classify samples into five categories using the molecular.subtyping function, and particularly luminal A, luminal B, HER2− enriched, basal-like and normal-like.

Using VST-normalized counts, sample-specific pathway scores were measured using a Gene set variation analysis (GSVA) as implemented in the GSEApy package (https://github.com/zqfang/GSEApy). The pathway-level annotation was defined according to the Hallmark gene set (n=50 pathways) from the Molecular Signatures Database (MSigDB). For finding pathways significantly differently expressed between CDKPredict low- and high-risk cases, a Mann-Whitney U test corrected for family-wise error (using the Benjamini-Hochberg procedure with a false discovery rate < 0.05) was employed. The correlation between CDKPredict risk scores and the pathway-level activity was carried out using a Spearman’s correlation analysis with significance tested using the Benjamini-Hochberg procedure.

For defining estrogen low, medium and high tumors, we utilized the HALLMARK_ESTROGEN_RESPONSE_EARLY gene set from the Hallmark collection, representing a biological transcriptional output in response to the activation of the estrogen-receptor signaling pathway^54^. Utilizing sample-specific GSVA, we observed a trimodal probability density function, which was modelled using a three component GMM to define three-distinct groups using the Brent’s method, as described above for finding CDKPredict risk-group cutoffs.

For assessing the relative enrichment of the estrogen response early pathway activity in low-risk compared with high-risk tumors, we conducted a gene set enrichment analysis (GSEA) employing the GSEApy package, employing genes ranked according to the normalized enrichment score: sign(log2[Fold change]) × − log10(qvalue) as calculated by a differential gene expression analysis conducted with DESeq2 (https://github.com/scverse/PyDESeq2) using VST normalized counts, using the CDKPredict low-risk group as a reference.

## Supplementary Material

This is a list of supplementary files associated with this preprint. Click to download.

- Supplementarytable03.xlsx
- Supplementarytable01.xlsx
- Supplementarytable02.xlsx
- Supplementarytable08.xlsx
- Supplementarytable04.xlsx
- Supplementarytable13.xlsx
- Supplementarytable05.xlsx
- Supplementarytable11.xlsx
- Supplementarytable06.xlsx
- Supplementarytable09.xlsx
- Supplementarytable07.xlsx
- Supplementarytable12.xlsx
- Supplementarytable10.xlsx

## Extended Data

**Extended Data Fig. 1.**
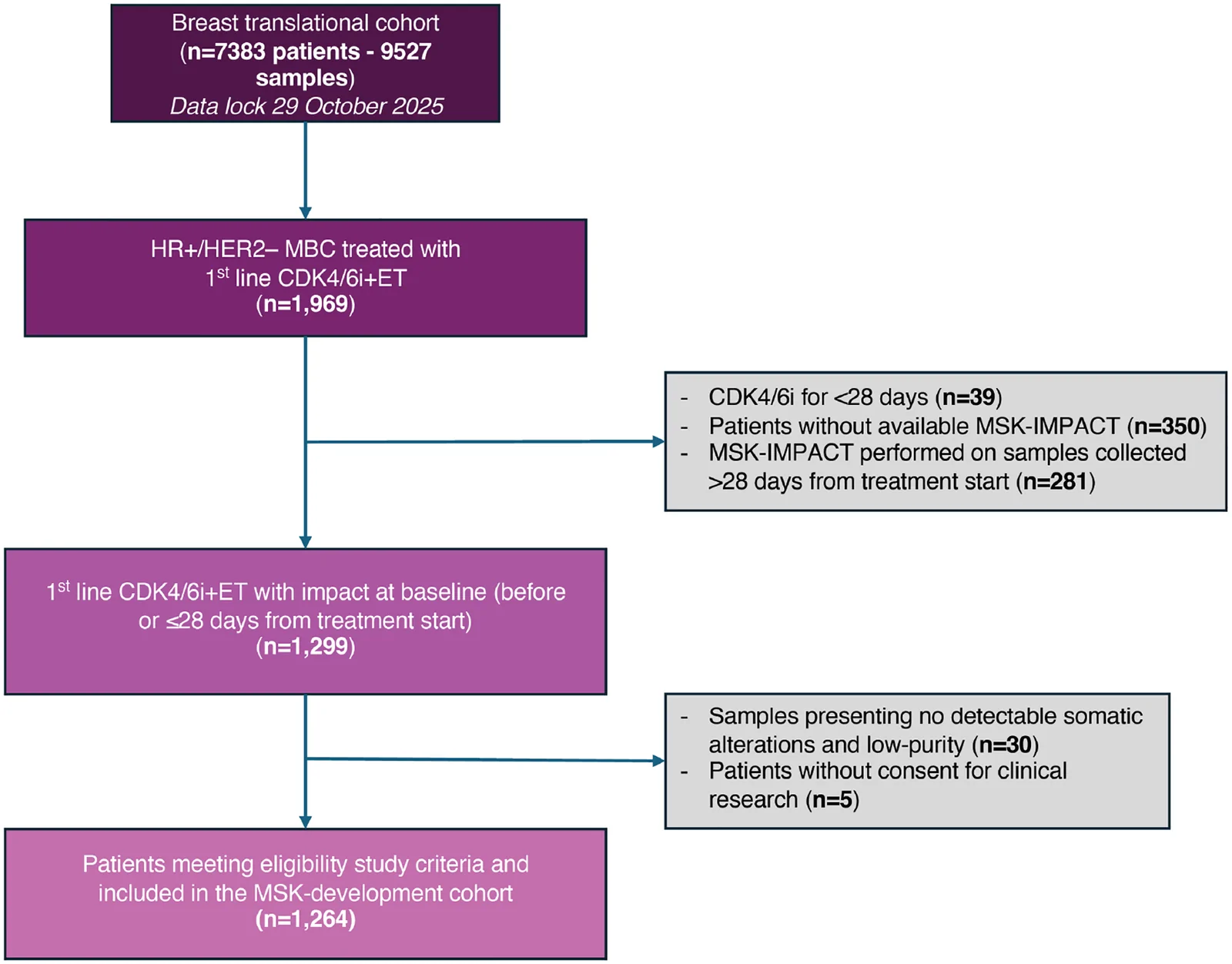
Consort diagram of the MSK-cohort. Consort diagram demonstrating inclusion and exclusion criteria of individuals with breast cancer included in the Memorial Sloan Kettering Breast Translational Dataset for the definition of the development cohort. Ultimately, 1,264 patients affected by HR+/HER2− breast cancer who received first-line CDK4/6i+ET together with baseline tumor-genomic sequencing were included in the study population.

**Extended Data Fig. 2.**
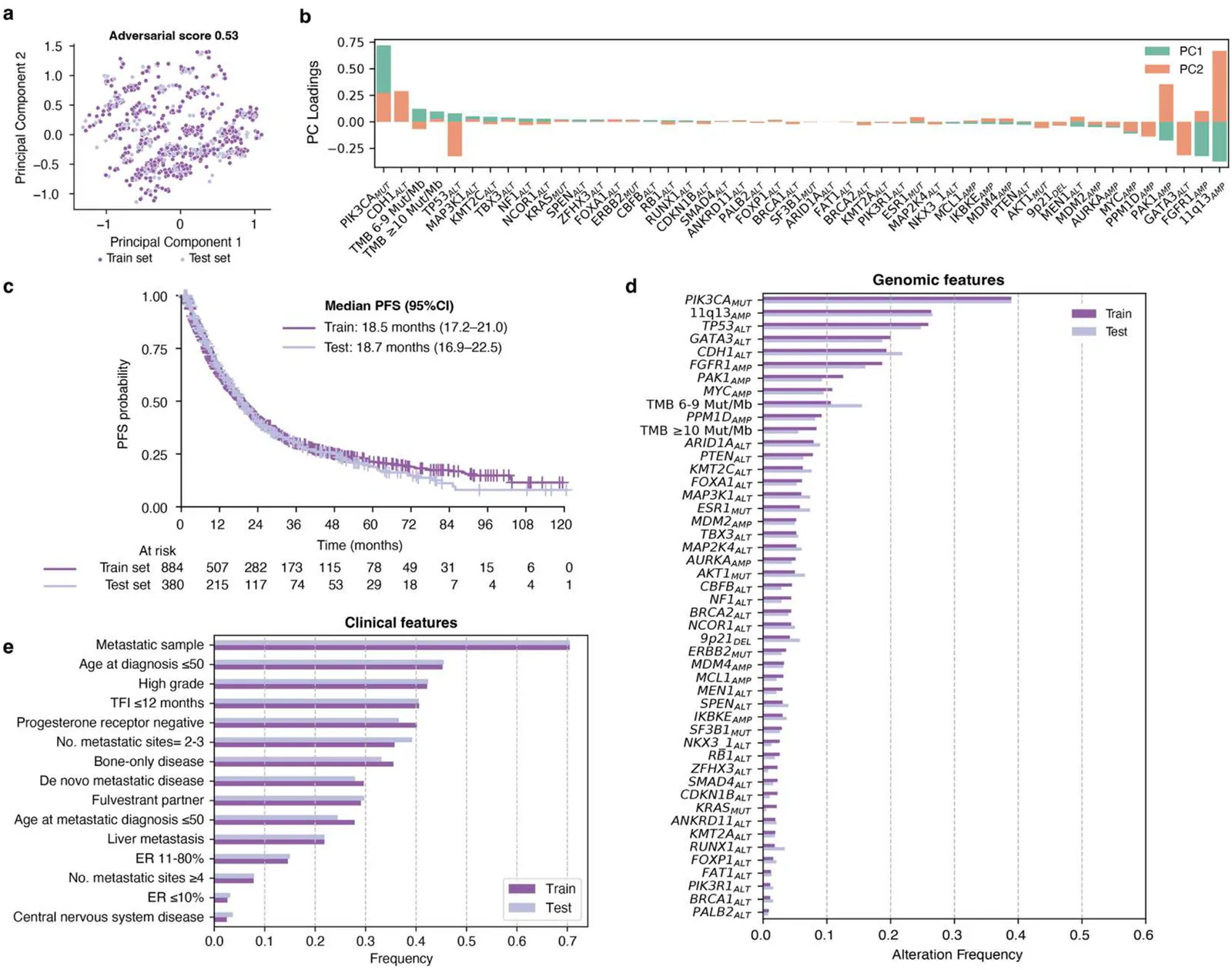
Comparison of genomic and clinical features between the training and the holdout MSK validation set. **a**, Principal component analysis between the training and the internal holdout validation set in the MSK-cohort using genomic variables and the n=2 principal components, showing an overall overlapping high-dimensional embedding of genomic features (Adversarial score of a random forest classifier of 0.53, Methods). **b**, Principal component loadings showing the contribution of the original genomic features in the definition of the n=2 principal components (Methods). **c**, Clinical outcomes on first-line CDK4/6i+ET between the training and validation set. **d**, Frequency of genomic alterations between the training and MSK-validation set. **e**, Same, using clinical features.

**Extended Data Fig. 3.**
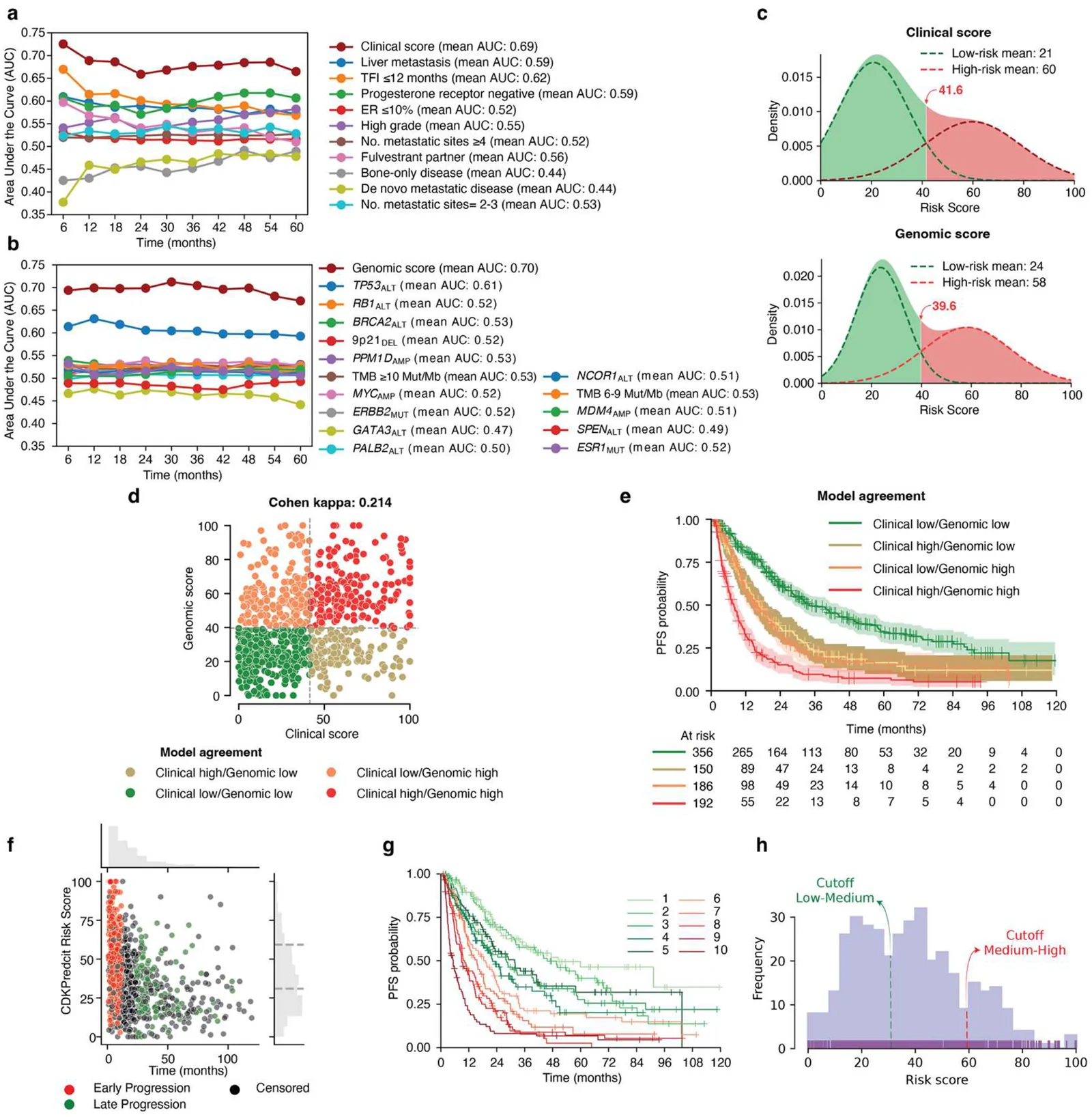
Clinical validity of the prediction model. **a**, Comparison of the time-dependent AUC at the 60-month landmark analysis including clinical risk scores and standalone clinical variables showing a significant association with PFS (not corrected for family wise error, *P*<0.05). **b**, same as above but using genomic-risk scores and standalone genomic variables, showing significant genomic variables as determined with a univariable Cox-regression corrected for family-wise error (*q*<0.05 by Benjamini-Hochberg procedure), **c**, Representation of the probability density function using a 2-component Gaussian-mixture model for defining low- and high-risk tumors in the clinical (*above*) and genomic (*bottom*) model. **d**, Correlation between genomic and clinical risk scores, showing an overall low agreement between the clinical and genomic risk estimation. **e**, Clinical outcomes according to the level of agreement between the two models, showing a stark improved baseline risk stratification compared with standalone models. **f**, Correlation between clinicogenomic risk scores and PFS on CDK4/6i+ET in the training set, showing a progressive shorter time to progression with increasing risk scores. Cases showing a PFS event before 12 months were colored in red, cases showing a PFS event ≥24 months in green, and censored patients in black. The top histogram denotes the time distribution, while the right histogram denotes the distribution of clinicogenomic risk-scores. **g**, Association between continuous risk-scores of the clinicogenomic model and PFS on CDK4/6i+ET in the training set, categorized in n=10 quantile bins. **h**, Risk-score distribution of patients included in the hold-out validation set related to the cutoffs between risk-strata established in the training cohort, showing a clear data-driven separation of the 3-risk groups to emphasize the plausibility of model’s classifications.

**Extended Data Fig. 4.**
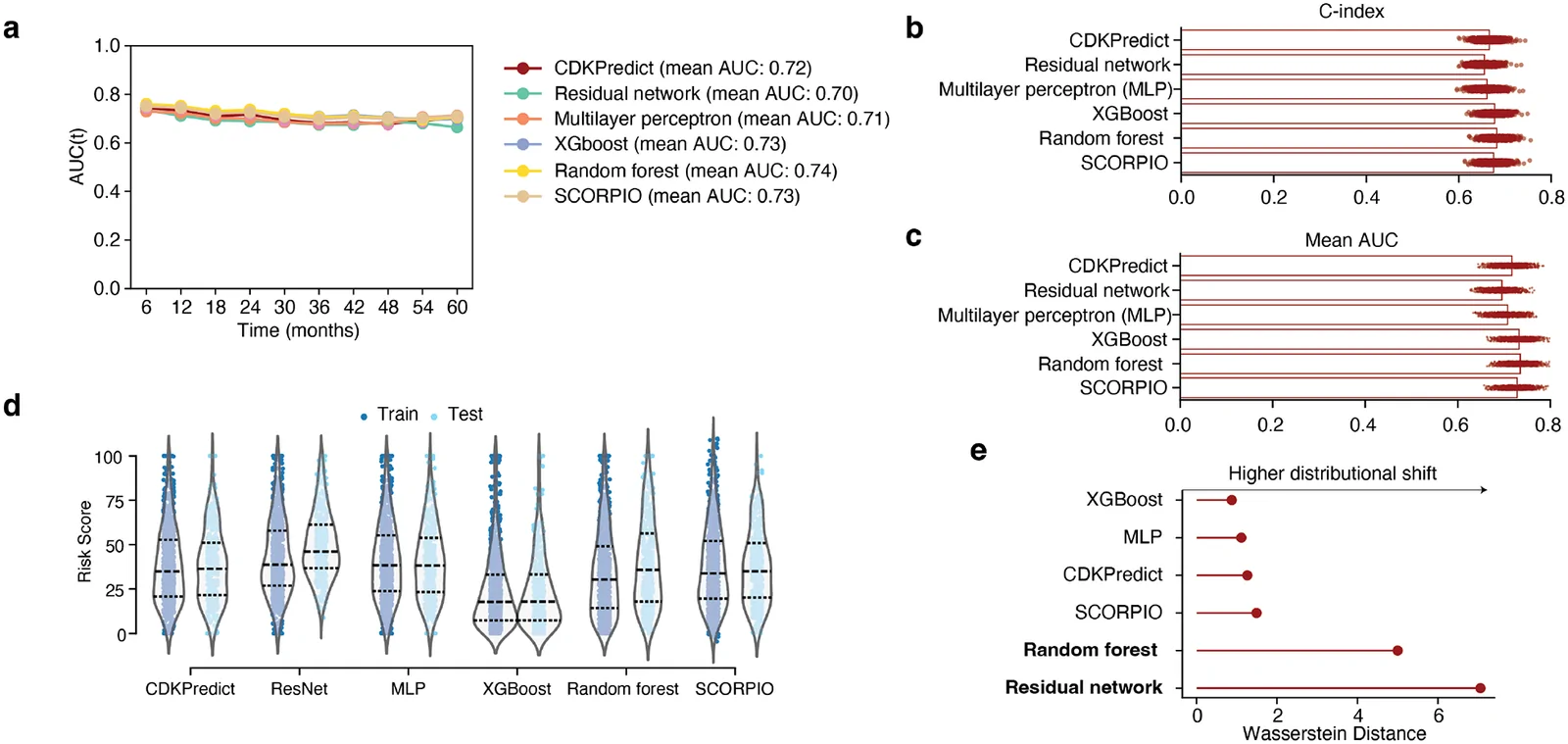
Benchmark of CDKPredict to state-of-the-art machine learning architectures. **a**, Comparison of the 5-year time-dependent AUC between comparator models and CDKPredict in the MSK-holdout validation set (n=380 cases), showing overlapping discriminative prediction at the advantage of a higher clinical-interpretability of CDKPredict. **b**, Comparison of C-index obtained across n=1,000 bootstrap reiterations of the validation set, using the original models fitted on the training set and utilizing the same reproducible artificial resampling (random seeds) across the different models. **c**, Same using the mean time-dependent AUC at 5 years, showing the overall overlapping predictive discrimination across the tested models. **d**, Comparison in the distribution of risk-scores between the MSK training and hold-out validation set, with horizontal dotted lines showing the 25^th^, 50^th^, and 75^th^ quantiles from bottom to top, demonstrating robustness to avoid overfitting and data-shifts by CDKPredict given the comparable representation of clinical and genomic variables and similar clinical outcomes between the two sets. **e**, Measurement of the distributional shift of risk-scores between the training and the hold-out validation set, with lower Wasserstein distances denoting reduced distributional shifts and thus higher distributional stability, with models represented in bold showing a significant (*P-value* <0.05) difference in the cumulative distributions between the two sets as determined using a Kolmogorov-Smirnov test.

**Extended Data Fig. 5.**
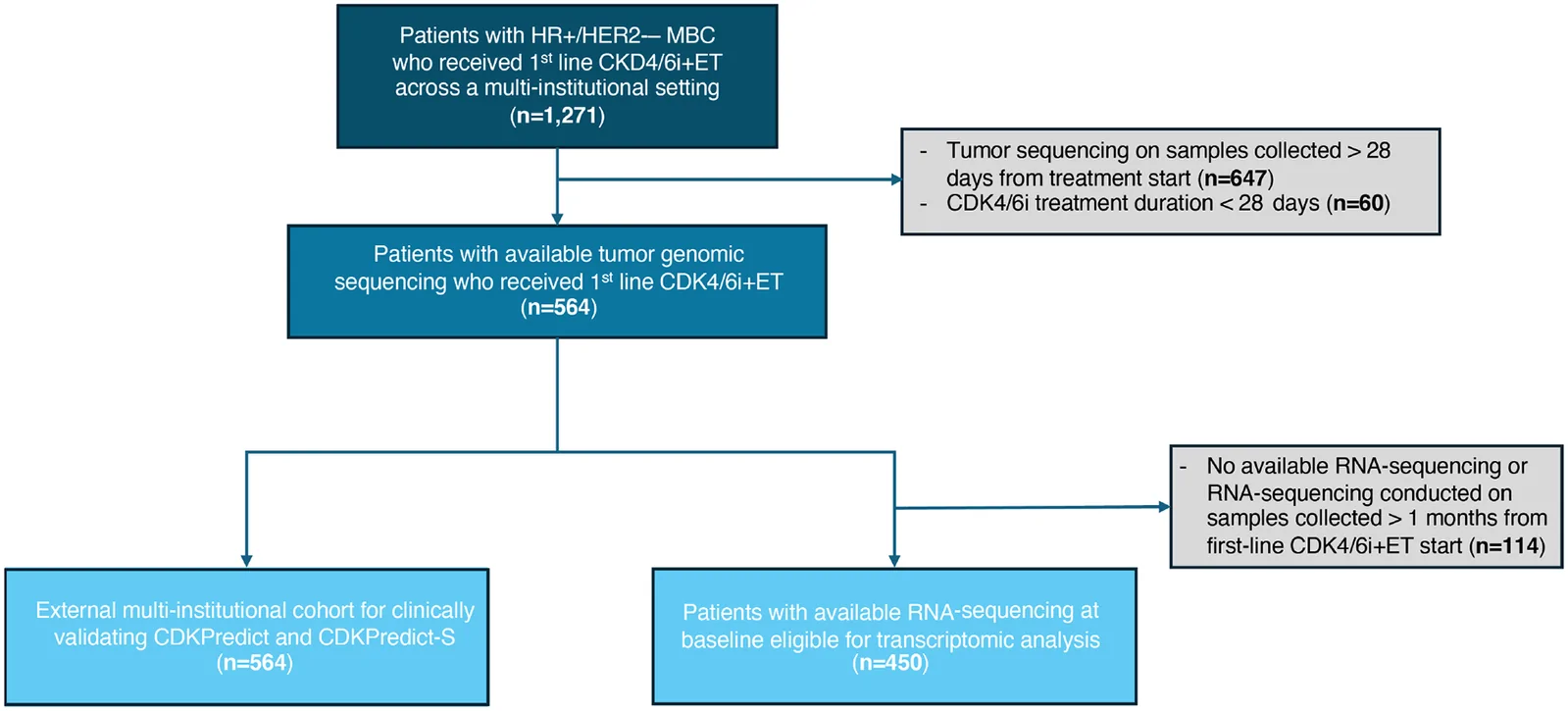
Consort diagram of the external multi-institutional cohort. Consort diagram representing inclusion and exclusion criteria for patients included in the external multi-institutional cohort employed for the independent clinical validation of CDKPredict. Ultimately, 564 individuals with breast cancer who received first-line CDK4/6i+ET and baseline tumor genomic sequencing were included for the clinical validation of the clinicogenomic model. Of them, 450 (79.8%) cases received baseline transcriptomic sequencing, and were considered for the transcriptomic analysis aimed at evaluating the biological plausibility of CDKPredict.

**Extended Data Fig. 6.**
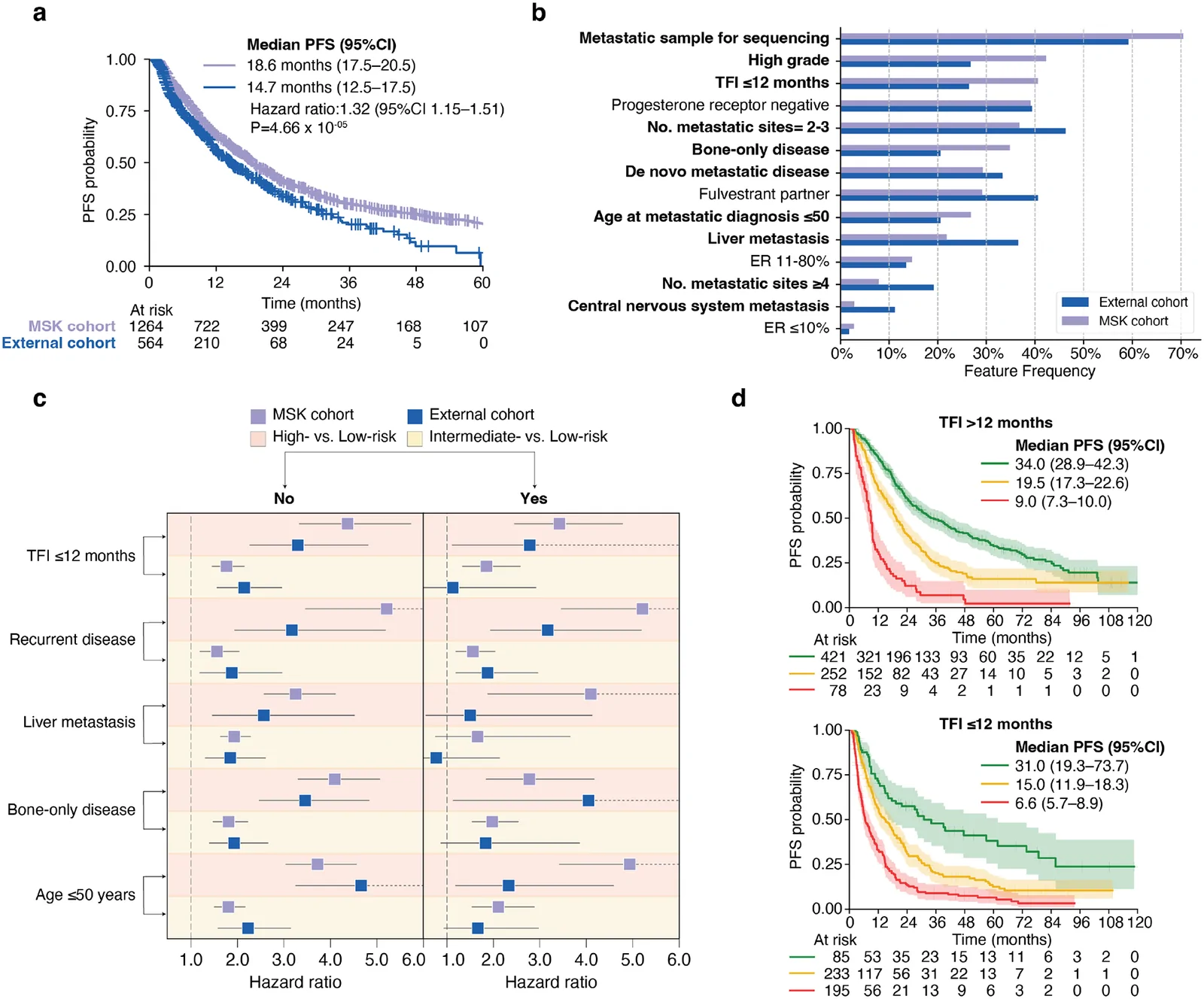
External multi-institutional cohort and clinical validation of CDKPredict across clinical relevant subgroups. **a**, Comparison of PFS on first-line CDK4/6i+ET between the MSK-cohort and external validation cohort. **b**, Distribution of clinical variables between the overall MSK-cohort and the external validation set, with labels in bold representing clinical variables showing a significantly different frequency between the two datasets (*q*<0.05 by Benjamini-Hochberg procedure). **c**, Subgroup analysis demonstrating the validity of CDKPredict across clinically relevant subgroups separately in the external and MSK-cohort, with the red and yellow rows demonstrating the hazard ratio of high-risk and intermediate-risk cases, respectively, relative to the low-risk group, utilized as reference, emphasizing the generalizability of the model across the overall population with breast cancer considered for first-line CDK4/6i+ET. **d**, Clinical outcomes according to CDKPredict risk stratification relative to a treatment-free interval above (*top*) and equal or below 12 months (*bottom*), recapitulating the clinical observations from the external test cohort in the larger MSK-population.

**Extended Data Fig. 7.**
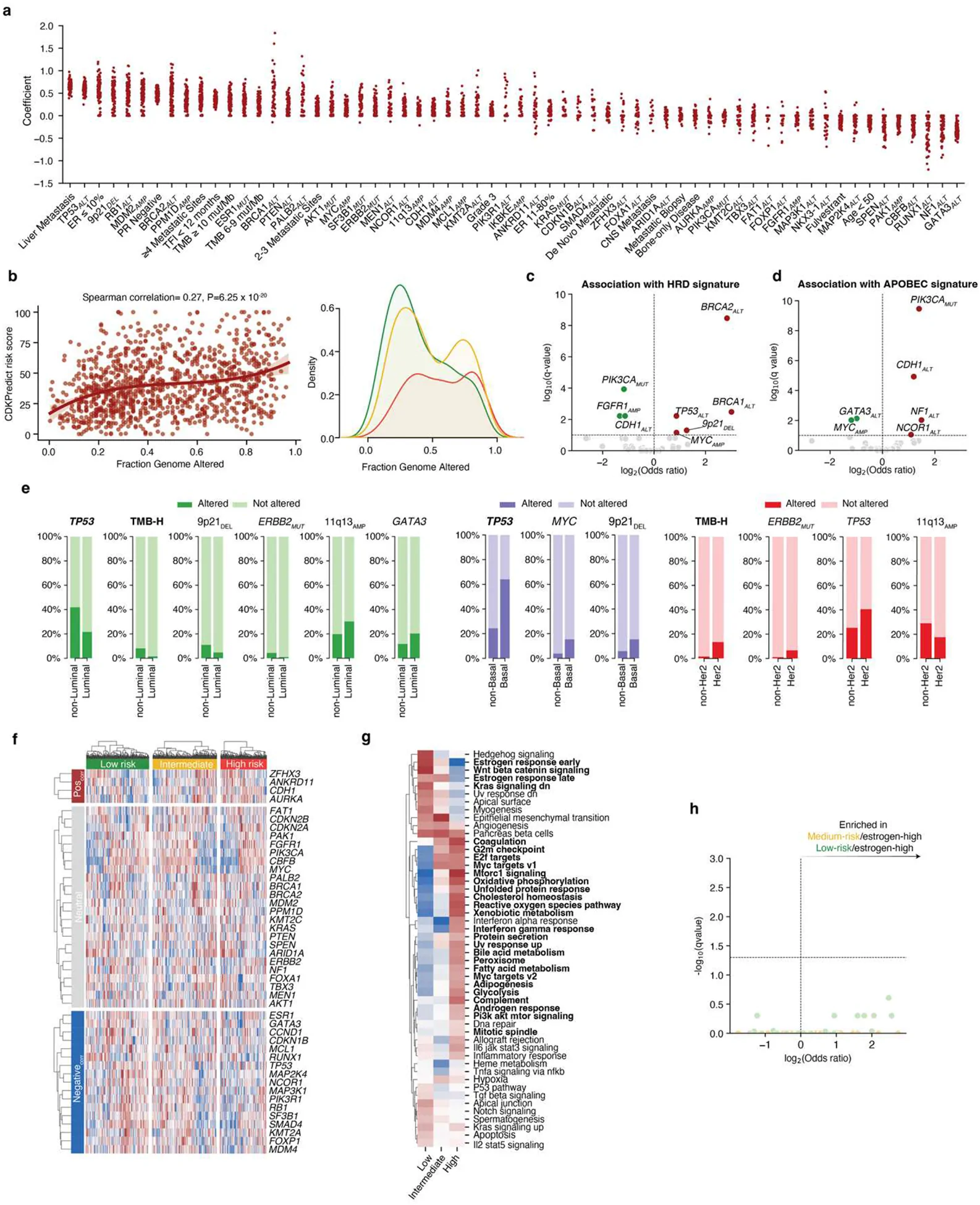
Plausibility of CDKPredict. **a**, Distribution of Cox-coefficients across the n=100 base learners included in the ensemble clinicogenomic model. **b**, Correlation between CDKPredict risk scores and fraction genome altered (FGA; *left*), showing a positive correlation resulting in a distinct distribution of FGA across risk groups, with a bimodal distribution observed prominently among intermediate- and high-risk tumors (*right*). **c**, Gene alteration enrichment analysis in HRD-dominant compared with non-HRD dominant tumors, calculated using the Fisher’s exact test and significance tested using the Benjamini-Hochberg procedure. **d**, Same, analyzing gene alterations enrichment in APOBEC-dominant compared with non-APOBEC dominant tumors. **e**, Co-segregation of genomic features included in CDKPredict and transcriptomic PAM50 intrinsic subtypes, representing genes showing an overall enrichment or mutual exclusivity in the relative subtype as calculated by a Fisher’s exact test (*P*<0.05), with genomic variables in bold showing genes with a statistically significant association once corrected for family wise error (*q*<0.05 using the Benjamini-Hochberg procedure). **f**, Heatmap showing the gene expression profile of features included in CDKPredict across risk-strata and categorized according to the Spearman’s correlation (not corrected for family-wise error, *P*<0.05) in genes showing a positive, non-significant, and negative correlation with clinicogenomic risk-scores. **g**, Representation of pathway-level activity across risk-strata, calculated as the mean expression for each group utilizing the Hallmark gene set collection; pathways represented in bold denote those with a significant different expression between low- and high-risk tumors, calculated using the Mann-Whitney U test and significance using the Benjamini-Hochberg procedure (*q*<0.05). **h**, Gene enrichment analysis in CDKPredict intermediate-risk and low-risk tumors showing a high Hallmark estrogen response early activity compared with the non-estrogen response early high/CDKPredict risk counterparts.

**Extended Data Fig. 8.**
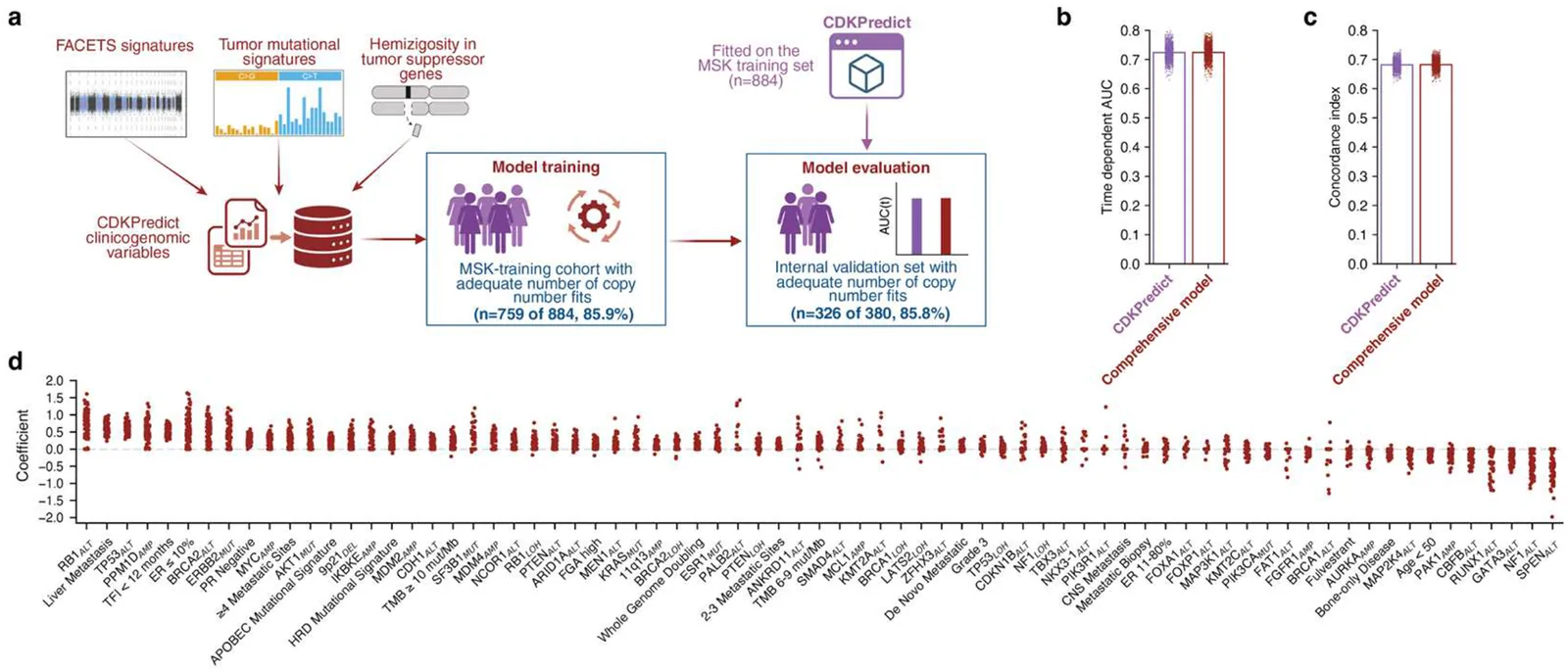
Benchmarking of CDKPredict to an augmented model including higher-order genomic features. **a**, Model development schema; in detail, beside features included in CDKPredict, the augmented model included FACETS signatures (fraction genome altered and whole genome doubling events), tumor mutational signatures (dominant status), and hemizygosity in tumor suppressor genes showing a significant prognostic association with PFS on CDK4/6i+ET (Methods). Thereafter, the model was fitted on the training set using the same CDKPredict architecture and tested in the independent MSK-validation set. **b**, Time dependent AUC using a 5-year timeframe between CDKPredict and the comprehensive model, with dots showing the mean AUC across n=1,000 bootstrap iterations in the validation cohort obtained using the same fitted model, using the same reproducible artificial splits between the two models (random seed). **c**, Same as before using the Harrel’s C-index score; altogether, the two models demonstrated similar performances, emphasizing the adoption of CDKPredict at the advantage of higher generalizability. **d**, Representation of features coefficients across the n=100 base learners included in the comprehensive model.

**Extended Data Fig. 9.**
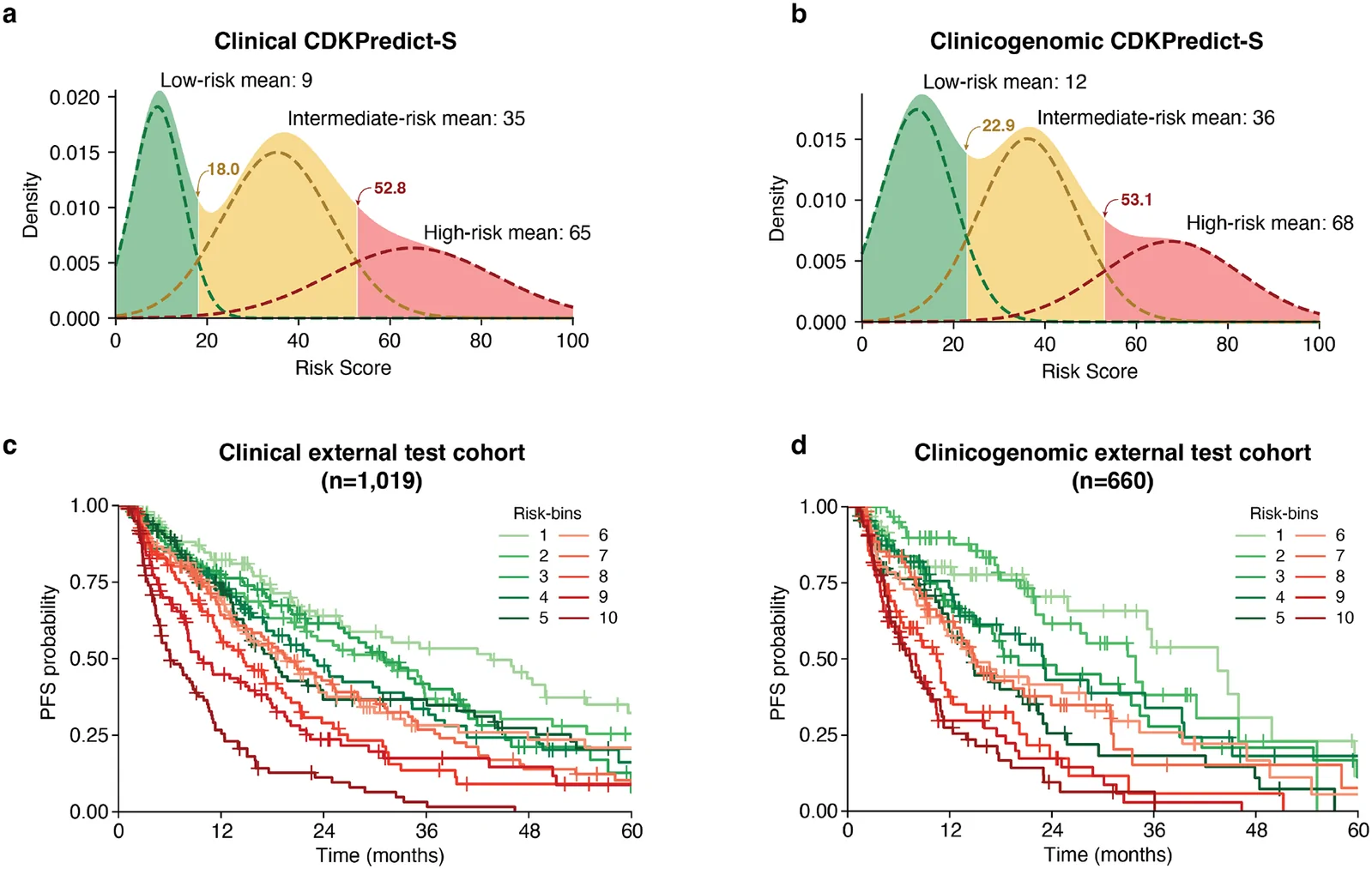
Deployment and assessment of the clinical and clinicogenomic version of CDKPredict-S. **a**, Probability density function of risk-scores across risk-strata of the clinical CDKPredict-S, which was refitted on the overall MSK-cohort before model deployment. Arrows and the respective values represent the risk-score cutoff between groups, calculated using the Brent’s method. **b**, Same for the clinicogenomic CDKPredict-S. **c**, Association between continuous risk-scores of the clinical CDKPredict-S and PFS on first-line CDK4/6i+ET in the overall independent external cohort with available clinical data (n=1,019), showing a clear stepwise association between increasing risk-scores and shorter time to progression. **d**, Same, using the clinicogenomic model in the external validation cohort of patients with available baseline tumor genomic sequencing (n=660).

## Figures and Tables

**Fig. 1.**
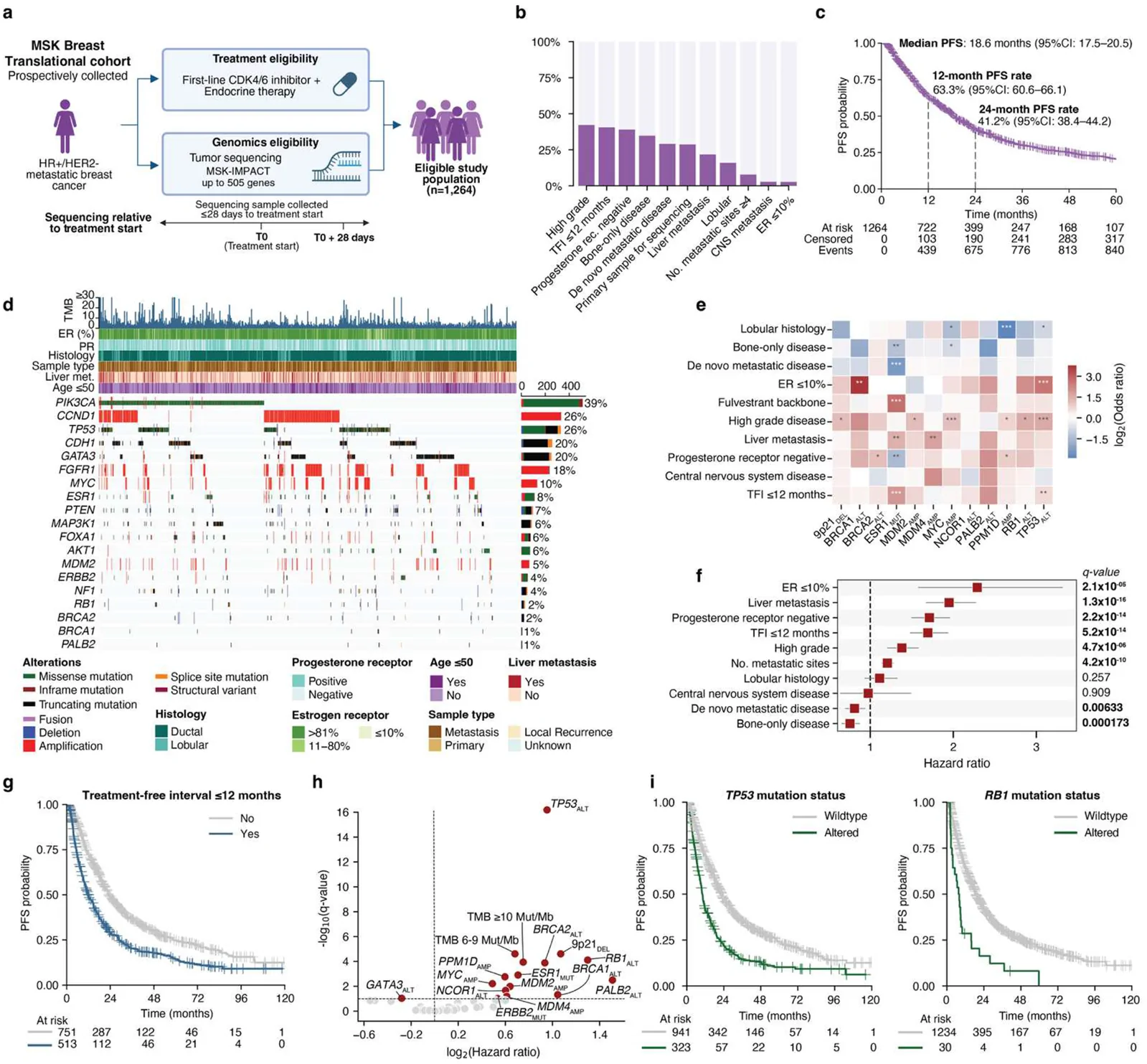
Clinical and genomic prognostic biomarkers for first-line CDK4/6i+ET. **a**, Representation of the MSK-cohort, encompassing 1,264 individuals with breast cancer who received first-line CDK4/6i+ET, together with tumor genomic sequencing at baseline. **b**, Distribution of clinical variables in the MSK-cohort. **c**, Median PFS on first-line CDK4/6i+ET in the MSK-cohort, together with the 12-month and 24-month PFS rate. **d**, Genomic landscape of the MSK-cohort, with the top annotation showing the co-segregation with relevant clinical features. **e**, Association between clinical and genomic variables in the MSK-cohort, demonstrating expected associations measured using the Fisher’s exact tests and log_2_(odds-ratios), with significance defined using the Benjamini-Hochberg procedure (* denotes q-value <0.05, ** q-value <0.01, *** q-value <0.001). **f**, Univariable Cox-regression between clinical variables and PFS on first-line CDK4/6i+ET, showing the effect size and statistical significance (adjusted for family-wise error using the Benjamini-Hochberg procedure). **g**, Clinical outcomes according to currently adopted criteria of high-risk in clinical trials investigating escalated first-line therapeutic options in HR+/HER2− MBC, namely TFI ≤12 months. **h**, Prognostic genomic alterations for first-line CDK4/6i+ET, demonstrated with a univariable Cox regression with significance determined using the Benjamini-Hochberg procedure. **i**, Clinical outcomes according to *TP53* (left) and *RB1* mutation status (right).

**Fig. 2.**
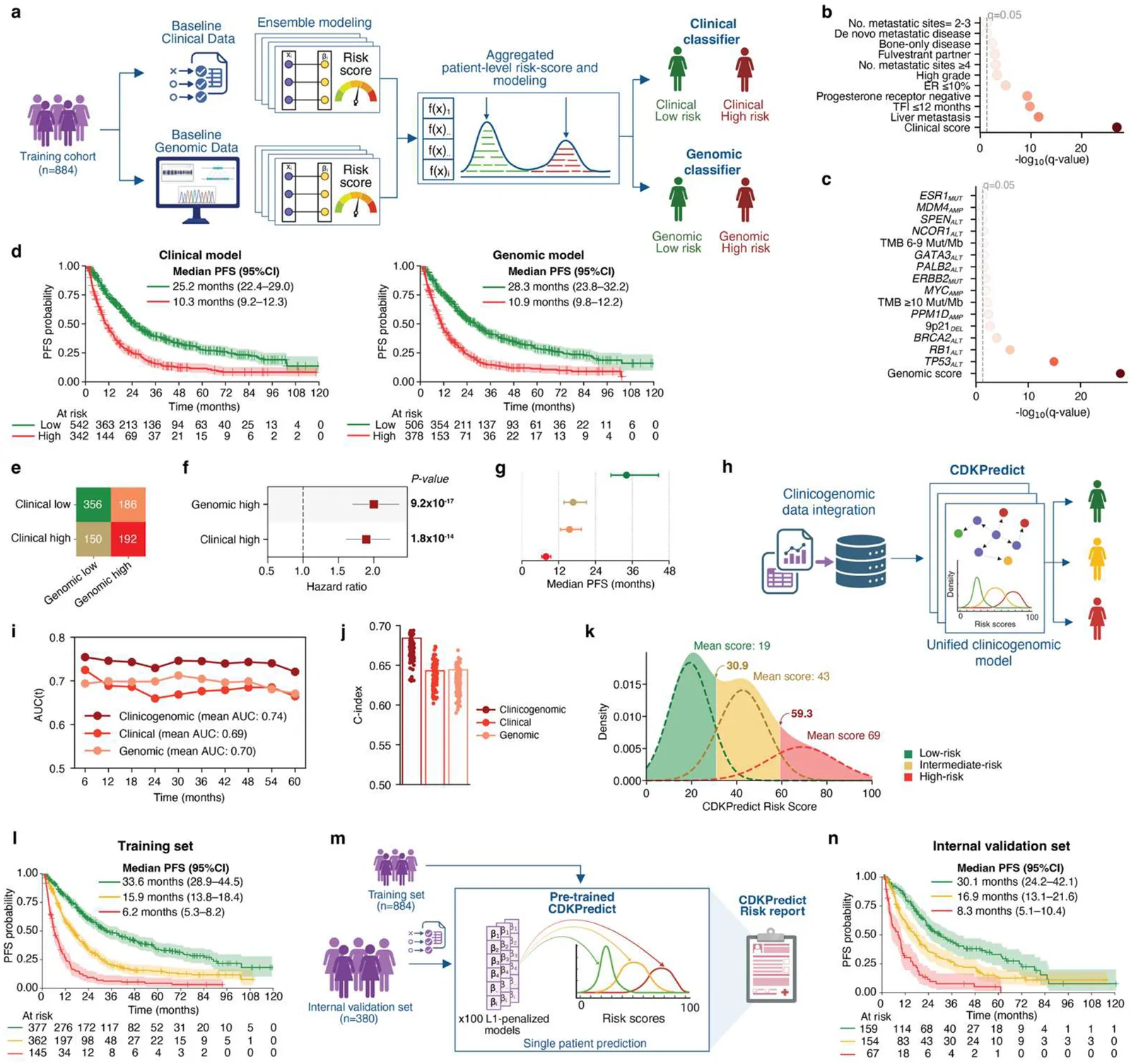
Development of a clinicogenomic model designed for baseline risk stratification on first-line CDK4/6i+ET. **a**, Development strategy of a clinical-only and genomic-only model to predict patients at high-risk of early progression to first-line CDK4/6i+ET. **b**, Univariable Cox-regression using clinical variables included in the clinical model and the clinical risk scores, with significance defined using the Benjamini-Hochberg procedure. **c**, Same as above, but showing significant genomic variables (*q*<0.05) and the genomic risk scores. **d**, Clinical validity of the clinical (left) and genomic (right) classifiers in the MSK-training set. **e**, Concordance between the clinical and genomic classifiers shown with a confusion matrix, demonstrating only moderate agreement between the two models (Cohen’s kappa=0.214). **f**, Multivariable Cox-regression including genomic and clinical risk-groups, demonstrating the non-overlapping prognostication between the clinical and genomic risk estimation. **g**, Forest plot showing the median PFS and 95% confidence intervals of risk-strata according to the level of agreement between the two classifiers. **h**, Development of a clinicogenomic model integrating both clinical and genomic variables to result in a 3-risk strata classification. **i**, Time-dependent AUC comparing the performance of the three models, integrating or not clinicogenomic variables. **j**, C-index between the 3 independent classifiers. The bar value demonstrates the final C-index of the ensemble model, and the dots demonstrate the C-index of the n=100 base learners encompassed within each ensemble architecture. **k**, Definition of 3-risk groups by the clinicogenomic model using a 3-component Gaussian mixture model, displaying the probability density function of the three components and cutoffs, these latter defined using the Bren’s method. **l**, Clinical validity of the clinicogenomic model in the training set. **m**, Following development, the model was independently tested on n=380 (30%) patients who received routine care at MSK (holdout internal validation set). **n**, Assessment of the clinicogenomic model in the holdout internal validation set.

**Fig. 3.**
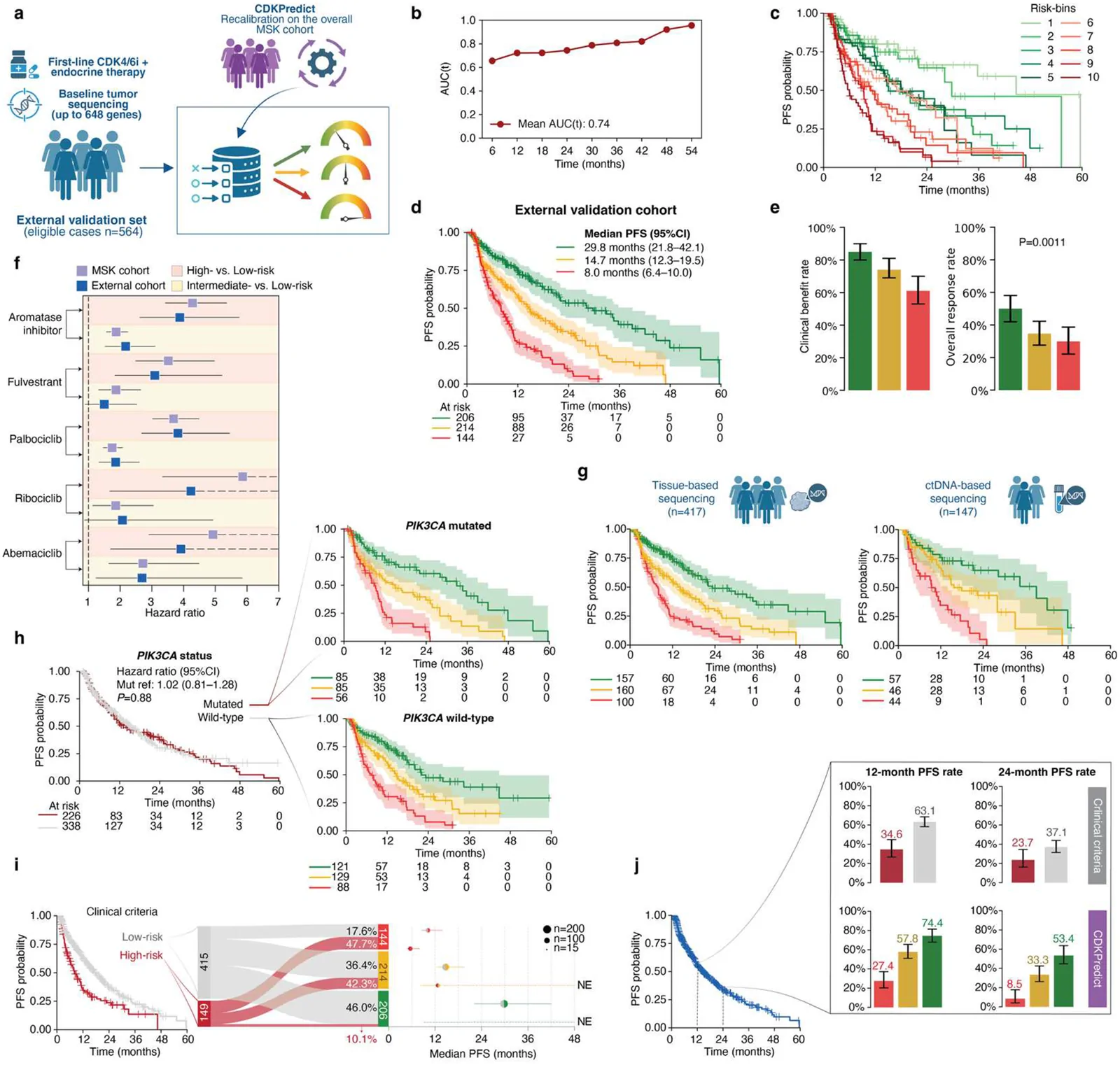
Independent clinical validation of CDKPredict in an external multi-institutional cohort. **a**, Workflow of the clinical validation of CDKPredict, which fine-tuned on the overall MSK-cohort, was tested using an external multi-institutional cohort of patients who received first-line CDK4/6i+ET and tumor sequencing at baseline (n=564 cases). **b**, Time-dependent AUC, with the 54-month to 60-month timeframe missing due to the available follow-up in the external cohort (median follow-up by reverse Kaplan-Meier method: 17.7 months [IQR 9.1–30.9] vs. 50.4 months [IQR 25.8–79.2] in the MSK-cohort). **c**, Association between continuous clinicogenomic risk-scores and median PFS, showing reduced time-to progression with increasing risk scores (divided in 10-quantile bins). **d**, Clinical validity of CDKPredict in the external cohort, showing robust and clinically meaningful baseline risk stratification. **e**, Disease control at 6-month (*left*) and overall response rate by radiological evaluation (*right*), with significance tested using the Pearson’s chi-square test. **f**, Clinical validity of CDKPredict across FDA-authorized compounds, with each bin (red and yellow) demonstrating the univariable Cox-hazard ratio of the high (red) and medium (yellow) risk groups compared with the low-risk category, utilized as reference. **g**, Performance of CDKPredict across distinct sequencing platforms, with model classifications retaining robust clinical validity for tumors sequenced using solid- and liquid-based panels. **h**, Clinical validity of CDKPredict according to *PIK3CA* mutation status. **i**, Sankey plot showing the level of agreement between historic clinical criteria of high-risk (TFI ≤12 months) and CDKPredict classifications, with the resulting progression-free survival according to the level of agreement demonstrated in the corresponding forest plot (*right*). **j**, 12-month and 24-month progression-free survival according to historic clinical criteria of risk and CDKPredict classifications, demonstrating the superior clinical validity of the clinicogenomic model.

**Fig. 4.**
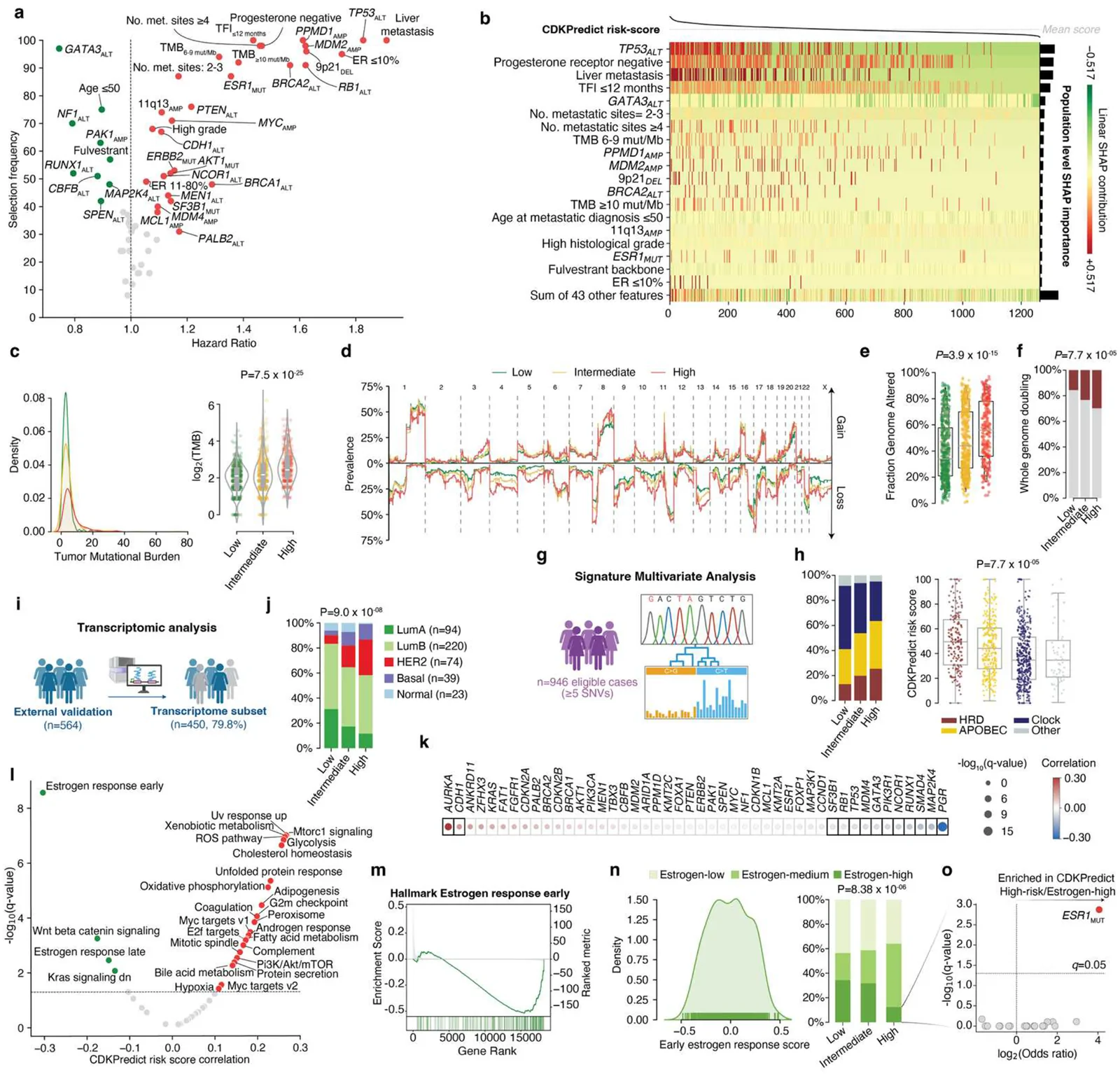
Clinical and biological plausibility of CDKPredict. **a**, Feature importance across the CDKPredict framework, with the selection frequency by base learners displayed on the y-axis, and the mean effect size on the x-axis. **b** Interpretable features contribution for CDKPredict risk-score using Shapley additive explanations (SHAP); colors denote the linear contribution of features in the risk-score estimation, with the overall population-level importance by SHAP values shown by the histogram on the right; the top annotation demonstrates the overall CDKPredict risk-scores of predicted samples, sorted in descending order. **c**, Probability density distribution (*left*) of TMB and log_2_-scale distribution (*right*) across risk-groups in the MSK-cohort, with significance calculated using the Kruskal-Wallis test. **d**, Genome-wide copy-number profile across risk-strata, showing an overall increasing burden of copy number alterations in higher risk-classes. **e**, Boxenplot of fraction genome altered across risk groups, with significance calculated using the Kruskal-Wallis test. **f**, Whole-genome doubling events across risk-strata, with significance calculated using the Pearson's chi-square test. **g**, Assessment of tumor mutational signatures using SigMA for evaluable tumors carrying at least n=5 single-nucleotide variants (n=946, 74.8%). **h**, Representation of dominant tumor mutational signatures across risk groups (*left*), and CDKPredict risk scores across dominant tumor mutational signatures (*right*). **i**, Study schema for the transcriptomic analysis, including patients from the external cohort who received whole-exome transcriptomic sequencing at baseline (n=450 of 564, 79.8%). **j**, PAM50 intrinsic subtypes across CDKPredict risk-strata, with significance determined using the Pearson’s chi-square test. **k**, Correlation between clinicogenomic risk-scores and RNA expression of variables included in CDKPredict, calculated using the Spearman’s correlation, with significance measured with the Benjamini-Hochberg procedure, with black boxes denoting significant correlations (*q*<0.05). **l**, Spearman’s correlation between clinicogenomic risk scores and pathway-level activity utilizing the Hallmark gene set collection. **m**, Gene-set enrichment analysis for genes included in the estrogen response early Hallmark set, with gene rank calculated using the normalized enrichment score from a differential gene-expression analysis between low- and high-risk tumors (using DESeq2; normalized enrichment score=−1.90, *q*=0.0010). **n**, Probability density distribution of the Hallmark estrogen response early pathway in the study population, with estrogen-high tumors showing a relative enrichment among lower risk classes. **o**, Gene enrichment analysis in CDKPredict high-risk/estrogen-high compared with CDKPredict high-risk/non estrogen-high tumors, measured with a Fisher’s exact test.

**Fig. 5.**
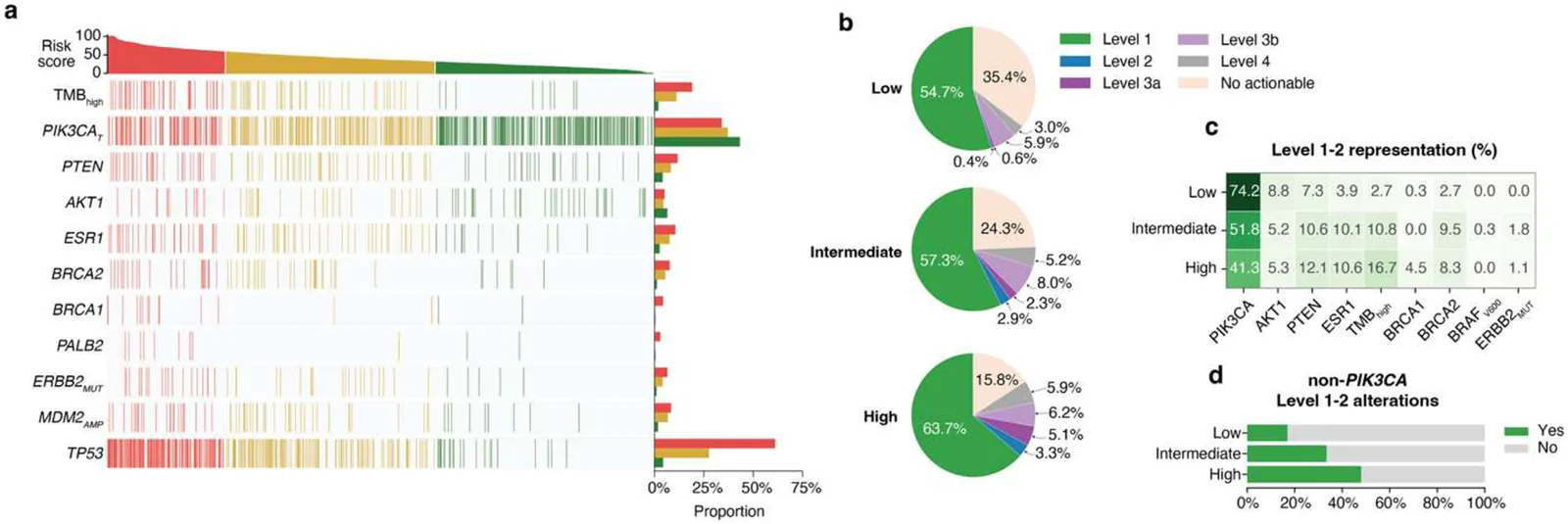
Actionable targets across CDKPredict risk-classes. **a**, Oncoprint of variables included in CDKPredict with relevant actionability in breast cancer, with the top annotation demonstrating CDKPredict risk-scores in decreasing order, and the right barplot showing feature frequency across CDKPredict risk classes. **b**, Overall representation of the highest-level of actionability per patient across risk-strata, as annotated in OncoKB, an FDA-Recognized Human Genetic Variant Database; overall, a higher prevalence of actionable alterations was observed across higher-risk groups. **c**, Representation of level 1 and level 2 alterations across risk-strata, including as the denominator the frequency of level 1–2 alterations per risk-group, showing the higher representation of additional standard-of-care actionable targets among higher risk-groups. **d**, Level 1 and level 2 alterations once excluding *PIK3CA* alterations across CDKPredict risk-groups.

**Fig. 6.**
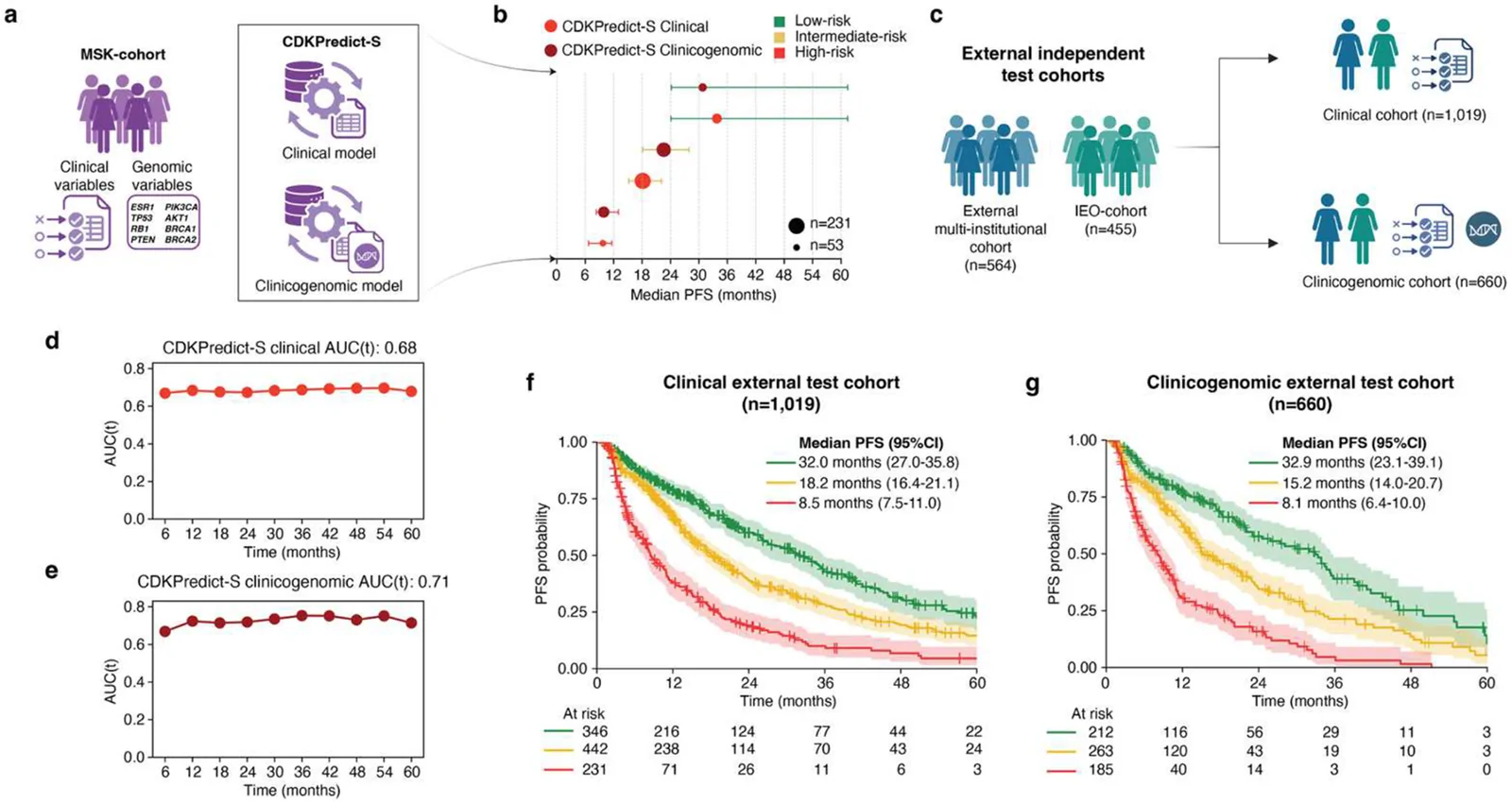
Development and clinical validation of CDKPredict-S. **a**, Framework for developing CDKPredict-S, a scalable model intended for variable resource settings, using a restricted subset of variables to facilitate its broad clinical implementation. **b**, Performance of CDKPredict-S in the hold-out MSK validation set (n=380), with the forest plot showing the median PFS and the confidence intervals according to CDKPredict-S clinical (*orange*) and CDKPredict-S clinicogenomic (*dark red*) risk-estimation. **c**, External independent clinical validation of CDKPredict-S across two external cohorts of patients treated with first-line CDK4/6i+ET, with or without available genomic sequencing at baseline. Overall, 1,019 patients were available for the clinical validation of the clinical model, while 660 patients received baseline tumor-sequencing and were included for the clinical validation of the clinicogenomic model. **d**, Time dependent AUC of the clinical model in the pooled external validation cohort (n=1,019). **e**, Time dependent AUC of the clinicogenomic model in the pooled external validation cohort of patients with available baseline genomic sequencing (n=660). **f**, Clinical validation of the clinical CDKPredict-S, demonstrating a statistically significant and clinically meaningful separation of outcomes according to the baseline risk stratification. **g**, Same, showing the clinical validation of the clinicogenomic model in patients with available genomic sequencing at baseline.

## Data Availability

Deidentified data needed to replicate our study findings are provided as Supplementary Tables. In accordance with Tempus and institutional policies of IEO, all requests for data collected or curated in-house will be evaluated on a case-by-case basis to determine whether the data requested and the use case comply with intellectual property and patient privacy obligations.

## Code availability

The custom code used for developing CDKPredict and CDKPredict-S and the code used for conducting the study analysis will be made available at https://github.com/lboscolobielo/CDKPredict.
